# Supplementary material for: Loophole-free Bell inequality violation with superconducting circuits
Source: Nature. 2023 May 10;617(7960):265–70. doi: 10.1038/s41586-023-05885-0 (PMC10172133; doi:10.1038/s41586-023-05885-0)
Supplement: Supplementary file 1 — This Supplementary Information file contains 11 sections, Supplementary Figs. 1–12, Tables 1–6 and additional references. [file 41586_2023_5885_MOESM1_ESM.pdf]

nature portfolio

https://doi.org/10.1038/s41586-023-05885-0

Supplementary information

Loophole-free Bell inequality violation with superconducting circuits

In the format provided by the authors and unedited

Supplementary Information
Loophole-free Bell Inequality Violation with Superconducting Circuits

Simon Storz,[1,*] Josua Schär,[1] Anatoly Kulikov,[1] Paul Magnard,[1,†] Philipp Kurpiers,[1,‡] Janis Lütolf,[1] Theo Walter,[1] Adrian Copetudo,[1,§] Kevin Reuer,[1] Abdulkadir Akin,[1] Jean-Claude Besse,[1] Mihai Gabureac,[1] Graham J. Norris,[1] Andrés Rosario,[1] Ferran Martin,[2] José Martinez,[2] Waldimar Amaya,[2] Morgan W. Mitchell,[3,4] Carlos Abellan,[2] Jean-Daniel Bancal,[5] Nicolas Sangouard,[5] Baptiste Royer,[6,7] Alexandre Blais,[7,8] and Andreas Wallraff[1,9,¶]

[1] *Department of Physics, ETH Zurich, 8093 Zurich, Switzerland*
[2] *Quside Technologies S.L., C/Esteve Terradas 1, Of. 304, 08860 Castelldefels (Barcelona), Spain*
[3] *ICFO - Institut de Ciencies Fotoniques, The Barcelona Institute of Science and Technology, 08860 Castelldefels (Barcelona), Spain*
[4] *ICREA - Institució Catalana de Recerca i Estudis Avançats, 08010 Barcelona, Spain*
[5] *Université Paris-Saclay, CEA, CNRS, Institut de Physique Théorique, 91191 Gif-sur-Yvette, France*
[6] *Department of Physics, Yale University, New Haven, CT 06520, USA*
[7] *Institut Quantique and Département de Physique, Université de Sherbrooke, Sherbrooke, Québec J1K 2R1, Canada*
[8] *Canadian Institute for Advanced Research, Toronto, Ontario M5G 1Z8, Canada*
[9] *Quantum Center, ETH Zurich, 8093 Zurich, Switzerland*

(Dated: April 20, 2023)

CONTENTS

I. Bell Inequalities, Tested Model and Underlying Assumptions 1

II. Random Basis Choice 3

III. Cryogenic System 6

IV. Quantum Devices 7

V. Experimental Setup 8
- A. Wiring Configuration 8
- B. Setup Synchronisation 8

VI. Characterization of State Preparation and Master Equation Simulations 10

VII. Qubit Readout 11

VIII. Comparison to Published Loophole-free Bell Tests 13
- A. Bell Violation, Repetition Rate and p-value 13
- B. Locality Loophole 14

IX. Phase Stability of Experimental Setup 16

X. Data Processing and Statistical Analysis 17
- A. Data Processing 17
- B. Calculation of p-value 17
- C. No-signalling 18

XI. Space-like Separation 19
- A. Critical Path 19
- B. Determining the Bell Time Budget 19
- C. Trigger Jitter 20
- D. Measurement of Protocol Duration 21

References 22

* simon.storz@phys.ethz.ch
† Current address: Alice & Bob, 75015 Paris, France
‡ Current address: Rhode & Schwarz, 81671 München, Germany
§ Current address: Centre for Quantum Technologies, National University of Singapore, 117543 Singapore
¶ andreas.wallraff@phys.ethz.ch

I. BELL INEQUALITIES, TESTED MODEL AND UNDERLYING ASSUMPTIONS

In its simplest form, a Bell test involves two distinct parties A and B, each holding a device delivering an outcome $x, y = -1$ or $+1$ when provided an input $a, b = 0$ or 1. The statistics generated in such an experiment can be described by a joint conditional probability distribution $P(xy|ab)$. Without loss of generality, this distribution can be refined in terms of additional (possibly unknown or "hidden") variables

$$P(xy|ab) = \sum_{\lambda} P(xy\lambda|ab). \tag{1}$$

For simplicity, we assume here that the hidden variables λ admit a finite set of possible values (over which the sum runs), but our derivation also holds for continuous sets of variables, or in fact any objects admitting a measure [1].

The key element of a Bell test is a Bell inequality, which constrains the statistics $P(xy|ab)$ that can be observed by the parties under a set of well-defined conditions. These conditions put constraints on the models which are tested in the Bell test, see below.

We consider the following two mathematical conditions. First, the *locality condition*

$$P(x|aby\lambda) = P(x|a\lambda) \tag{2a}$$
$$P(y|abx\lambda) = P(y|b\lambda) \tag{2b}$$

expresses the fact that the outcome x of A only depends on information that is available to A's device locally (similarly for y and B's device). Notice that the outcome of A's device may still depend on the additional variable λ in an unknown, arbitrary way. Unlike the inputs and outcomes, the variable λ may be shared between the parties and can carry an unlimited amount of information. Hidden variable models satisfying the locality condition are known as *local hidden variable models.*

Second, the *measurement independence condition*

$$P(\lambda|ab) = P(\lambda) \tag{3}$$

expresses that the additional variable λ, which can be understood as the state of the devices, is statistically independent from the settings a and b. Equivalently, this condition states that the settings a and b are chosen independently from the hidden variable λ.

We now show that the two conditions above are sufficient to guarantee the validity of Bell's inequalities. Indeed, Eq. (1) can be rewritten as

$$P(xy|ab) = \sum_\lambda P(\lambda|ab)P(xy|ab\lambda) \tag{4}$$
$$= \sum_\lambda P(\lambda)P(x|aby\lambda)P(y|ab\lambda) \tag{5}$$
$$= \sum_\lambda P(\lambda)P(x|a\lambda)P(y|b\lambda), \tag{6}$$

where we used the above two conditions as well as the chain rule of probability theory. The factorization of probabilities given by Eq. (6) characterizes local hidden variable models under the condition of measurement independence, and exactly defines the set of statistics which satisfy Bell's inequalities [2]. In particular, this factorization implies:

$$|S| = \left|\langle x\cdot y\rangle_{(0,0)} - \langle x\cdot y\rangle_{(0,1)} + \langle x\cdot y\rangle_{(1,0)} + \langle x\cdot y\rangle_{(1,1)}\right| \le 2 \tag{7}$$

where $\langle x\cdot y\rangle_{(a,b)}$ denotes the expectation value of the product of the individual measurement outcomes at sites A and B. This can be seen by defining the functions

$$\begin{aligned} X_a(\lambda) &= P(x=+1|a\lambda) - P(x=-1|a\lambda) \\ Y_b(\lambda) &= P(y=+1|b\lambda) - P(y=-1|b\lambda). \end{aligned} \tag{8}$$

When Eq. (6) holds, we then have

$$\langle x\cdot y\rangle_{(a,b)} = \sum_\lambda P(\lambda)X_a(\lambda)Y_b(\lambda), \tag{9}$$

and $S = \sum_\lambda P(\lambda)S(\lambda)$ can then be decomposed in terms of λ with each term satisfying

$$\begin{aligned} |S(\lambda)| &= |X_0(\lambda)(Y_0(\lambda) - Y_1(\lambda)) + X_1(\lambda)(Y_0(\lambda) + Y_1(\lambda))| \\ &\le |X_0(\lambda)(Y_0(\lambda) - Y_1(\lambda))| + |X_1(\lambda)(Y_0(\lambda) + Y_1(\lambda))| \\ &= |X_0(\lambda)|\,|Y_0(\lambda) - Y_1(\lambda)| + |X_1(\lambda)|\,|Y_0(\lambda) + Y_1(\lambda)| \\ &\le |Y_0(\lambda) - Y_1(\lambda)| + |Y_0(\lambda) + Y_1(\lambda)| \\ &= 2Y_0(\lambda) \\ &\le 2. \end{aligned} \tag{10}$$

Here, we used the triangle inequality, the condition $|X_a(\lambda)|, |Y_b(\lambda)| \le 1$ and supposed that $Y_0(\lambda) \ge Y_1(\lambda) \ge 0$ (the other cases can be treated similarly). This implies that $|S| \le \sum_\lambda P(\lambda)|S(\lambda)| \le 2$ as expected. We note that this conclusion holds equally for deterministic local hidden variable models (with $P(x|a\lambda), P(y|b\lambda) \in \{0,1\}$) and for non-deterministic ones.

This derivation of Bell's inequalities can be summarized in the following relation:

$$\left.\begin{array}{l}\text{locality condition}\\ \text{measurement independence}\end{array}\right\} \Rightarrow \text{Bell inequalities} \tag{11}$$

It implies that a violation of the CHSH Bell inequality (7) leads unequivocally to a contradiction with the locality or measurement independence condition as expressed in Eqs. (2)-(3), i.e. it rules out any local hidden variable model under the measurement independence condition.

In a practical Bell test, taking place in space-time rather than probability space, it may be hard to justify that a tested model should satisfy the above conditions, hence opening loopholes [3]. For instance, in a space-time configuration allowing for communication from A to B during a trial of the Bell test, A's setting choice a may become available at B's location, in which case the statistics produced by a hidden variable model may not satisfy Eq. (2), thus opening the *locality loophole*. Fortunately, this issue can be avoided by setting up a Bell experiment in an appropriate space-time configuration. To formalize this idea, we follow Bell's own analysis [4] and consider theories obeying the principle of local causality. In a *locally causal theory*, an event can only depend on the content of its past light cone. On the other hand, a theory that is not locally causal will be referred to as *non-local* (which does not imply signalling as discussed below). In order to test locally causal theories, we are interested in configurations where their accumulated statistics admit a local hidden variable model.

In the situation depicted in Fig. 4 of the main text, where the event at which A's output x is produced is space-like separated from the production of b and y on B's side, a local causal model doesn't allow x to depend directly on b and y. It can however depend on any other information present in its past light-cone. Hence, in a locally causal theory, x can always be expressed as a function $x(a, \mu)$, where a is A's setting choice and μ accounts for any additional information present in the past

light-cone of x that is useful in predicting x. Similarly, B's outcome y can be written in this configuration as the function $y(b,\tau)$, where τ is any information in the past light-cone of y that is relevant to predict y given b. Since the past light cones of x and y overlap, μ and τ can have some information in common, as well as some of their own. Gathering all this information together into a single variable $\lambda = (\mu,\tau)$, A and B's outcomes can be written as the functions $x(a,\lambda) = x(a,\mu)$ and $y(b,\lambda) = y(b,\tau)$, where τ and μ respectively are simply ignored. Therefore, the statistics of A's outcomes are of the form $P(x|a\lambda)$ and those of B's outcomes in the form $P(y|b\lambda)$, in agreement with the locality condition (2). This is only true, however, because of the space-like separation of the considered space time configuration. In particular, this conclusion requires an appropriate definition of the events at which the inputs a and b are created and those at which the outcomes x and y are fixed. Still, this shows that under the principle of local causality, the locality condition (2) transforms to an assumption on the time and location at which events happen.

Therefore, all locally causal theories produce correlations that satisfy the CHSH Bell inequality (7) when i) the event corresponding to a choice of setting of one party and measurement of the other party are space-like separated, ii) the measurement independence condition (3) holds. This derivation of Bell's inequalities in physical space can be summarized in the following relation:

$$\left.\begin{array}{l}\text{local causality}\\ \text{space-like separation}\\ \text{measurement independence}\end{array}\right\} \Rightarrow \text{Bell inequalities} \tag{12}$$

Note that some form of measurement independence condition is indeed needed here, because superdeterminism is both compatible with the requirements of local causality and able to produce a Bell inequality violation under space-like separation [3]. Formally, this assumption slightly reduces the power of the considered models: the devices can depend on any information contained in their past light-cone, except that which is used in their choice of settings. The amount of measurement independence between the choice of measurement settings and the tested local hidden variable models can however be arbitrarily small [5]. We discuss the choice of settings further in Supplementary Section II.

The relation Eq. (12) implies that a violation of a Bell inequality under the conditions of space-like separation and measurement independence invalidates the principle of local causality.

This conclusion can be phrased equivalently by stating that a violation of the inequality under the condition of space-like separation alone either invalidates the principle of local causality or the measurement independence condition. This option was relatively recently put forward as a way to address discussions of measurement independence [6].

We note here that no notion of realism appeared in our discussion so far, neither in the derivation of a Bell inequality, nor in the definition of the model. This is not to say that a Bell test could not be defined with such notions [11]. However, it shows that this concept is not necessary to interpret the result of a Bell test, as already noticed by Bell and others [1, 4, 7–10].

We emphasize that a violation of local causality does not imply faster-than-light communication and is therefore not in contradiction with special relativity. Indeed, quantum theory can violate Eq. (7) while satisfying the no-signalling conditions

$$P(x|ab) = P(x|ab') \tag{13a}$$
$$P(y|ab) = P(y|a'b), \tag{13b}$$

see [2] for more details.

Finally, let us comment on what *loopholes* [3] in Bell tests are. The *locality loophole* is open when either the choice of measurement settings or the measurement outcome of one party is available to the other party at the time of measurement. Space-like separation of the corresponding events at the two nodes closes this loophole. The *fair-sampling loophole* is considered to be open when a part of the observed statistics is discarded (on purpose or because of unwanted loss). Finally, the *memory loophole* is said to be open when the individual trials are assumed to be independent. All these loopholes were closed in the experiment reported in this manuscript.

II. RANDOM BASIS CHOICE

As explained in Supplementary Section I, a Bell test relies on the assumption of measurement independence, i.e., the condition that the basis choices (here a and b) are free parameters, i.e. not influenced by potential hidden variables that could determine the measurement outcomes [3]. This possibility cannot be ruled out: so-called superdeterministic local hidden variable models, in which all events including the setting choices are predetermined by initial conditions in the past, can be invoked to explain any possible experimental outcome [3]. The general consensus is therefore to support the assumption of measurement independence by choosing the measurement bases in a manner that is considered to be uncorrelated to all prior events, including potential hidden variables.

In this work, as in [12–15], we employ random number generators (RNGs) based on laser phase diffusion for choosing the measurement settings. As explained in [16], for the lasers and conditions used, measured phase diffusion rates imply that the laser phase is to a very good approximation uniformly distributed on the interval $[0,2\pi)$. We treat this phase as an ideal random variable. Interferometry, photodetection and digitization are used to produce a partially-random bit from this trusted random variable.

More precisely, the RNGs used in this work each employ eight quantum entropy sources (QESs), shown in

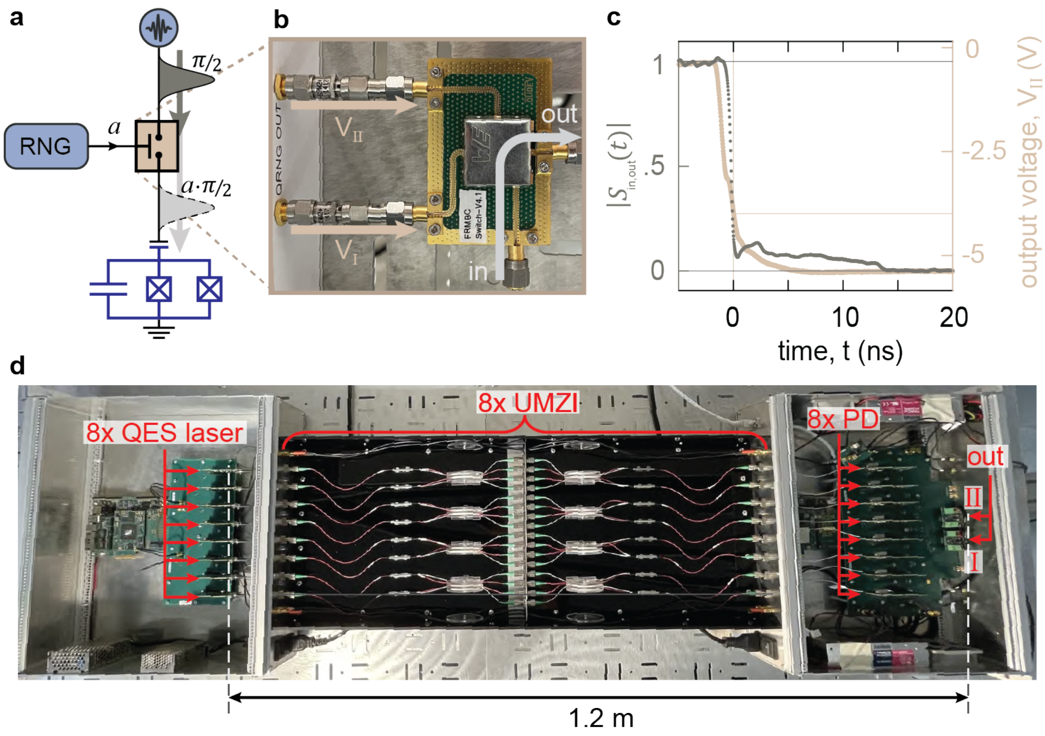

FIG. S1. Random basis choice. a, Scheme of a RNG output bit a controlling a microwave switch, which either blocks or transmits a $\pi/2$-pulse applied to the qubit drive. b, Photograph of the wiring from the RNG (left), its output ports V_I and V_{II} to the switch. The path of the microwave signal is displayed as a gray arrow. c, Output signal of the switch showing the signal amplitude versus time for a continuous microwave tone applied to the switch ($S_{in,out}$) and the trigger output voltage of the RNG, measured at port II (V_{II}). d, Photograph of the RNG with its eight QES, unbalanced Mach-Zehnder Interferometers (UMZIs) and photodiodes (PDs), used to generate a random number output at ports I and II, see text for details.

Fig. S1d, operated synchronously to generate in parallel eight raw random bits. The QES construction and operation are very similar to the RNGs described in [16], with the difference that the lasers are driven above and below threshold at a rate of 400 MHz and thus with a pulse repetition period of 2.5 ns. In each drive period, the current-modulated single-mode diode is kept below its lasing threshold for 1.5 ns, during which time the cavity field decays, and becomes highly susceptible to phase diffusion. The diode current is then taken above threshold for 1 ns. This produces a strong phase diffusion followed by the emission of a phase-randomized optical pulse. In this way a continuous, 400 MHz train of phase-randomized pulses is generated. These enter an unbalanced Mach-Zehnder interferometer (UMZI) that interferes each pulse with the preceding pulse, thus converting the phase-random pulse train into a power-random pulse train. This impinges on a photodetector (PD) that measures the signal power. The PD output is 1-bit digitized with a comparator, with its threshold adjusted to give equal probability of 0 or 1. A flip-flop and exclusive-OR (XOR) calculate the running parity of the comparator output, and this parity is the QES output bit. Within each RNG, fast logic computes the XOR, and thus the parity, of the simultaneously-generated raw bits from the eight QESs, to produce one output bit that is less predictable than any of the raw bits. This bit is output on two complementary output channels, as required to drive the microwave switch that follows.

The predictability of a bit X is quantified by the excess predictability ϵ, defined such that the probability of observing an output of zero, conditioned on all prior conditions, is $\frac{1}{2}(1-\epsilon) \leq P(X=0|\text{priors}) \leq \frac{1}{2}(1+\epsilon)$. As such, $(1+\epsilon)/2$ is an upper bound on the predictability, i.e. the maximum probability with which one could guess the result, knowing all prior conditions. $\epsilon = 0$ would mean that the bit is ideally random, and $\epsilon = 1$ would indicate a bit that may be fully predictable.

Due to noise stemming from the individual elements of the devices, the bits generated by the individual QESs can be partially correlated with pre-existing physical conditions, despite the unpredictability of the laser pulse's state. An agent who knows everything that can be known about the experiment prior to the operation of the QES, including all untrusted classical noise variables, but not the optical phase which we consider being fundamentally random, may be able to guess the output of the RNG according to the predictability ϵ.

In a first analysis (see Supplementary Section X), we assume the untrusted variables are, like the laser phase, independent of the hidden variables responsible for determining the measurement outcomes. Consequently, the

QES and RNG output bits are independent of those hidden variables, and from the perspective of the Bell test there is no excess predictability, i.e. $\epsilon = 0$.

To show that our result is nevertheless robust to a finite level of correlations between the hidden variable models tested and the state of the RNGs, in a second analysis (also in Supplementary Section X) we allow for non-zero excess predictability up to $\epsilon = 5.24 \times 10^{-6}$, which would include the value given by a validated physical model of the RNG system, described in [16]. This model uses signal and noise parameters measured on the individual QESs to estimate the excess predictability, including noise from the photodetector, digitizer, and delayed contributions of previous pulses. The resulting upper bounds for the individual QESs, with typical value $\epsilon_{\rm QES} \approx 0.2$, are shown in Table SI. We consider a model in which individual noise sources within a single QES may collaborate to maximize the predictability of the bit. We however assume that there is no exchange of information between the individual QES. This is a reasonable assumption as the current implementation is designed to avoid such cross-influences, and indeed no such cross-correlations were observable in measurements on the QESs comprising each RNG. Performing an XOR operation on the eight QES-generated raw bits then produces an exponential reduction in the resulting ϵ of the final bit. For this reason, we compute the excess predictability of the RNG as $\epsilon_{\rm RNG} = \prod_i \epsilon_{\rm QES,i} = 5.24 \times 10^{-6}$. We show in Supplementary Section X that this amount of excess predictability does not affect our conclusion. As a sanity check, we used the TESTU01 ALPHABIT randomness test suite [17] to look for statistical regularities in the setting choices for the single offset angle, high statistics experiment (see main text). These data passed all tests, indicating that no statistically significant regularities were found.

We note that earlier Bell test experiments that addressed measurement independence in a similar manner [12–15] had to make similar assumptions of the working of their device, i.e. not allowing for arbitrary cross-influences between the processes that generate different individual raw bits. Unless this limitation is imposed (which can be motivated on technical grounds), the excess predictability does not decrease when combining the output of several modules, as already shown by Santha and Vazirani [18]. This can be easily understood by considering just two modules producing bits a_1 and a_2. Since the modules are not space-like separated from each other, the classical information necessary to produce a_1 and a_2 from the individual uniform phases ϕ_{a_1} and ϕ_{a_2} can be considered jointly and denoted v_c. For the same reason, when the information about the phase ϕ_{a_1} reaches the first module, it is also available to the second module. Hence, when computing its output bit a_2, the second module has all necessary information, namely ϕ_{a_1} and v_c, to compute bit a_1 as well on its own. Therefore, a demon placed at module 2 could produce a bit a_2 that perfectly cancels the contribution from the first module

QES	ϵ	QES	ϵ
A_1	0.174	B_1	0.206
A_2	0.283	B_2	0.198
A_3	0.240	B_3	0.238
A_4	0.165	B_4	0.223
A_5	0.232	B_5	0.210
A_6	0.232	B_6	0.213
A_7	0.202	B_7	0.243
A_8	0.200	B_8	0.219
$\prod_i A_i$	$4.283\ 10^{-6}$	$\prod_i B_i$	$5.243\ 10^{-6}$

TABLE SI. Upper bounds on the excess predictabilities ϵ of each quantum entropy source (QES) of the RNG boxes at nodes A and B.

in the XOR $a_1 \oplus a_2$. The excess predictability of the combined bit in this case thus remains high at $\epsilon \approx 0.2$. Existing measurement dependent local hidden variable models taking advantage of excess predictability can reproduce CHSH values above $S = 2.7$ [19, 20].

The total time needed for the RNG to generate a random number is measured to be 17.10 ± 0.14 ns. This is the delay between the moment when the laser injection current crosses the lasing threshold and the moment when the RNG output voltage passes the threshold of $V_{\rm II} = -3.5\,$V for $a = 0$, as measured with a fast oscilloscope. The output $V_{\rm I}$ transitions simultaneously. As described in Refs. [16, 21], strong, spontaneous-emission-driven phase diffusion upon crossing the lasing threshold produces an effectively full randomization of the laser phase. For this reason, we take the injection current crossing threshold as the time of randomness generation. This is conservative (it overestimates the time between randomness generation and the output of the corresponding bit), because it does not include intra-laser delays such as the carrier number equilibration time and the time for the photon number to rise after crossing threshold. We note that this is the same methodology used to define and determine and the time for random bit generation in Refs. [12–14].

The measured delay of 17.10 ± 0.14 ns consists of $\sim 7.1\,$ns propagation delay of the laser pulses in the short optical path of the unbalanced Mach-Zehnder interferometer (UMZI), $\sim 3\,$ns for the conversion of output a to the control voltages $V_{\rm I/II}$, and $\sim 7\,$ns for other elements.

We have installed the optical fiber cables of the UMZI in a straight line along the axis of the cryogenic link of the Bell test experiment. This increased the total Bell distance by the separation between the box housing the QES and the RNG output port (1.2 m), providing an extra 4 ns for the Bell time budget.

To alternate between two measurement settings, we prepare a $\pi/2$ pulse to rotate the qubit measurement basis using an arbitrary waveform generator (AWG) and send it to a microwave switch. The switch operates in a band from DC to 18 GHz (CMD193C3 from Custom MMIC), and is used as a single-pole single-throw switch by terminating one of the output ports. The state of the switch is controlled by the differential voltage output of

the RNG. If the RNG returns bit $a = 0$, the voltage on port I is set to $V_{\rm I} = -5\,{\rm V}$ and on port II to $V_{\rm II} = 0\,{\rm V}$, leading to the switch blocking the microwave pulse, and vice versa for $a = 1$, which causes the switch to pass the signal. The RNG output voltage is maintained for the duration of a trigger sent from an AWG to the RNG. At the end of the trigger signal the RNG output is reverted to the default value of $a = 1$, or "switch open," thereby ensuring identical conditions at the beginning of each experimental trial. A scheme and a photo of the setup are shown in Fig. S1a, b. Figure S1c displays an example output voltage trace of a switching event. Here, the voltage measured at RNG output port II switches from 0 V to −5 V (with a small voltage offset) within approximately 1 ns. We record the generated random bit with an FPGA at each node to process its value together with the qubit readout result.

III. CRYOGENIC SYSTEM

To perform a non-local Bell test we have engineered, manufactured and assembled a distributed modular cryogenic system spanning a distance of 30 meters, see Fig. 3 in the main text. The system is composed of two dilution refrigerators connected via a cryogenic link with an additional pulse tube cooler positioned in the middle of the link. The cryogenic link consists of 2.5-m long modules. Each module contains a set of radiation shields cooled to temperatures of approximately 50 K, 4 K, 800 mK (still stage) and 15 mK (base temperature stage) housed in an o-ring-sealed vacuum can.

In order to successfully operate this cryogenic system, we considered three key aspects. First, the thermal load on each temperature stage has to be kept small compared to the cooling power available in the system. At high temperature stages (4 K and 50 K), the heat load on a stage is dominated by black-body radiation originating from outer layers. At low temperatures ($T < 4$ K) the heat load is dominated by conduction through mechanical support posts between the individual temperature stages. We minimized the radiation heat load by using multi layer insulation (MLI) at the 50K stage and by ensuring that each shield is radiation tight. We minimized conduction through support posts using a material with low thermal conductivity.

Second, it is essential to transport the heat efficiently along the different modules to the cryostats which provide cooling. For this purpose we used oxygen-free high-conductivity copper as a shield material.

Third, all copper radiation shields undergo thermal contraction of about −3.25 mm/m during the cooldown from room temperature to about 40 K (between 40 K and 15 mK, the amplitude of atomic vibrations in a lattice is significantly damped, and therefore the solid state does not undergo relevant thermal expansion or contraction) [22]. To compensate for this contraction we connect adjacent link modules using mechanically flexible

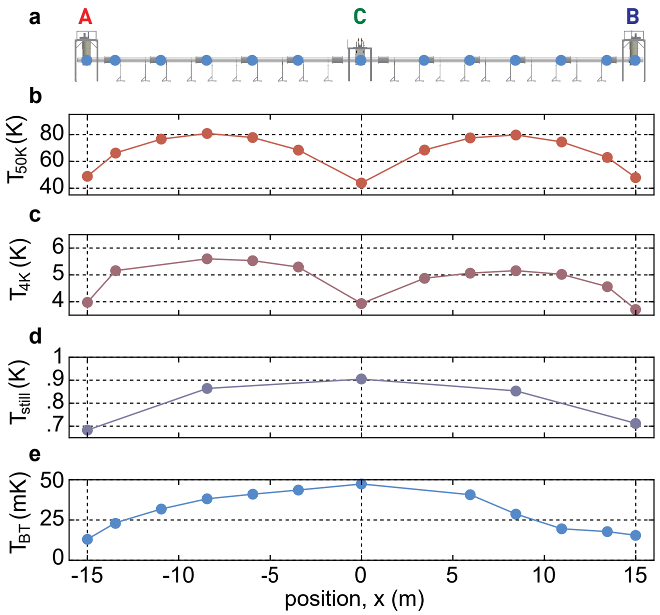

FIG. S2. (a), Scheme of the cryogenic system indicating the positions of temperature sensors along the axis of the link (blue dots). (b)-(e), Measured temperatures along the cryogenic system, 14 days after the start of the cooldown, at the (b) 50K, (c) 4K, (d) still and (e) base temperature stage. The lines are linear interpolations between the measured data points serving as a guide to the eye.

and highly conductive copper braids. The aluminium waveguide itself also undergoes thermal contraction of about −4.1 mm/m during the cooldown phase. For this reason we ensured it can slide freely across distances of several cm within the base temperature shield, while still being thermalized to the base shield with flexible copper braids.

The temperatures of different stages of the cryogenic link 14 days after the start of the cooldown are shown in Fig. S2. For the 50K and 4K stages the lowest temperatures are measured at the three cryostats, where pulse tube coolers actively extract heat. The highest temperatures (~ 80 K at the 50K stage and ~ 5.5 K at the 4K stage) were measured at the points which are the most distant from the cooling units. On the two low temperature stages (still and base), the heat load from the upper stages across the link is much lower (especially the radiative heat load, which scales with $\propto T^4$) than the cooling power available at the two outer nodes. It is therefore sufficient to cool these stages with the dilution refrigerators at the ends of the link. We measure base temperatures at both ends of around 15 mK, which is comparable to the temperatures measured in standard dilution refrigerators ($10 - 20$ mK). Along the link the base temperature is below 50 mK everywhere, see profile in Fig. S2, and the superconducting aluminium waveguide reaches temperatures below 60 mK.

The WR90 aluminium waveguide used as the quan-

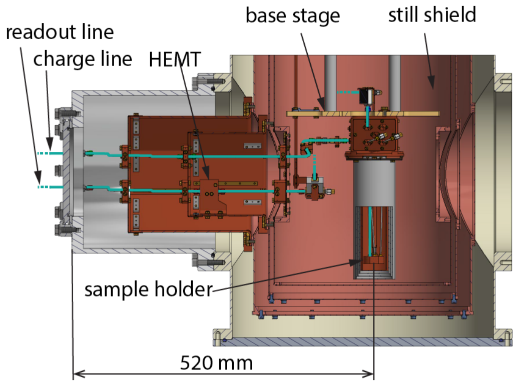

FIG. S3. CAD drawing (side view) of a custom-made cryogenic side-port and its RF cable routing. Two RF lines (light blue) are fed into the Bluefors LD dilution refrigerator for random basis choice and qubit readout.

tum channel between the two chips is housed within the base temperature shield. The loss of the superconducting microwave waveguide was measured to be about $1-2$ dB/km in the operating frequency range of $8-10$ GHz. For the measurement of the loss we used the method described in Ref. 23, but probing the waveguide in reflection with the far end terminated by a superconducting short. This measurement was conducted in a test system with a length of 21 m. As the results are consistent with the ones presented in Ref. 23 and 24, we estimate that the loss per meter is about the same in the present 30-m-long system. Overall, the loss in the channel is dominated by the insertion loss of the circulator (13%; see Fig. S5) and of the copper coaxial microwave cables connecting the waveguide to the samples ($\sim 3\%$ each).

In dilution refrigerators, microwave cables are usually routed vertically from the top of the vacuum can to the sample housed below the base temperature plate, and thermalized at each intermediate temperature stage [25]. For the Bluefors LD400 cryostats used in our experiments, this corresponds to a total cable length of around 1200 mm, adding a significant signal propagation delay to the overall time budget of the Bell test protocol. Therefore, to minimize signal propagation delay, we route the qubit drive input and readout signal output lines horizontally through a custom-built side-port, see Fig. S3. It reduces the overall cable length between the sample and room temperature to about 500 mm. In this way the signal propagation time between the room temperature electronics and the sample is reduced by 7.1 ns. In addition, the side-port increases the Bell distance (Supplementary Section XI) between $\vec{r}_{\text{start}}$ and $\vec{r}_{\text{end}}$ by 2×500 mm while a conventional vertical cabling would not. This increases the available time budget by 3.3 ns. In total, the side-port access requires 10.4 ns less of the time budget compared to a conventional routing scheme, which corresponds to roughly 3 meters of link length. The side-port contains radiation shields at the same temperature stages as the cryostat, and the RF cables are thermalised at each of the corresponding plates.

IV. QUANTUM DEVICES

We fabricated two samples with a size of 4.3 mm $\times$ 7 mm on silicon substrates. For each device, shown in Fig. S4, the coplanar waveguide structures and the qutrit pad were patterned in a 150-nm-thick niobium film on the substrate in a photolithographic process using reactive ion etching. The Al/Ti/Al airbridges connect ground planes along resonators and transmission lines to establish a well-defined ground at microwave frequencies. The airbridges were fabricated with a two-layer resist photolitography process. In a third step, we used electron-beam lithography and double-angle shadow evaporation to fabricate the Josephson junctions. For mounting, we glued and bonded the sample onto a copper printed circuit board, and packaged it in a copper sample holder. At each end of the link one sample holder was then mounted on the base temperature stage of the dilution refrigerator.

For the described experiment we fabricated two nominally identical samples. We characterized the parameters of the resonators, qutrit, and qutrit-resonator couplings for each device at 10 mK. The resonance frequencies, coupling rates and dispersive shifts of the readout and transfer resonator and Purcell filter circuits were determined from their transmission spectra with the transmon qutrit initialized in $|g\rangle$ and $|e\rangle$, using methods described in Ref. 26. We characterize the qutrit transition frequencies and coherence times using Ramsey-type experiments. In Table SII we list all measured quantities. We measure the properties of the qutrit and transfer circuitry with the qutrits tuned to their respective sweet-spot frequencies, which is the frequency they are operated at throughout

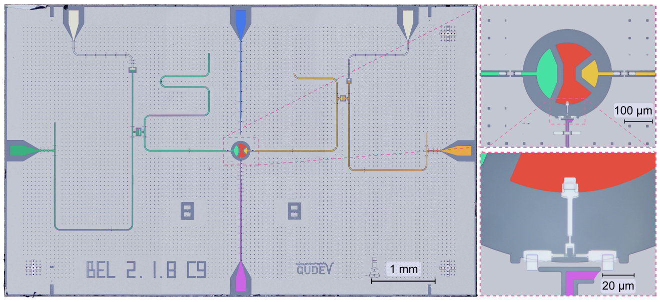

FIG. S4. False colour micrograph of one of the two samples used in the experiment, which are equal in design. The superconducting transmon qutrit is located in the center of the device (red), coupled to a microwave drive line (blue), a flux control line (purple), the readout circuitry (green) and the transfer circuitry (yellow). The insets show an optical microscope image of the qutrit and of the area around the SQUID loop of the device.

quantity, symbol	unit	Node A	Node B
qubit ge transition frequency, $\omega_{ge}/2\pi$	GHz	7.811	7.875
transmon anharmonicity, $\alpha/2\pi$	MHz	-311	-308
e to g level energy relaxation time, $T_{1\mathrm{ge}}$	μs	19.4	11.3
f to e level energy relaxation time, $T_{1\mathrm{ef}}$	μs	3.4	4.1
coherence time on ge subspace , $T^*_{2\mathrm{ge}}$	μs	7.3	6.9
coherence time on ef subspace, $T^*_{2\mathrm{ef}}$	μs	4.3	4.8
thermal excitation at equilibrium, n_{th}	%	6.9	8.3
$\lvert f,0\rangle \leftrightarrow \lvert g,1\rangle$ transition frequency, ν_{f0g1}	GHz	5.794	5.926
readout resonator frequency, $\omega_r/2\pi$	GHz	5.632	5.636
readout Purcell filter frequency, $\omega_{\mathrm{Pr}}/2\pi$	GHz	5.620	5.611
readout resonator/qutrit coupling, $g_r/2\pi$	MHz	198	224
readout circuit dispersive shift, $\chi_r/2\pi$	MHz	-6.4	-8.0
readout resonator/filter coupling, $J_r/2\pi$	MHz	29.9	26.2
readout Purcell filter bandwidth, $\kappa_{\mathrm{Pr}}/2\pi$	MHz	95.4	92.1
readout resonator eff. bandwidth, $\kappa_r/2\pi$	MHz	35.2	29.8
transfer resonator frequency, $\omega_t/2\pi$	GHz	9.490	9.491
transfer Purcell filter frequency, $\omega_{\mathrm{Pt}}/2\pi$	GHz	9.489	9.477
transfer resonator/qutrit coupling, $g_t/2\pi$	MHz	255	324
transfer resonator/filter coupling, $J_t/2\pi$	MHz	36.2	36.4
transfer Purcell filter bandwidth, $\kappa_{\mathrm{Pt}}/2\pi$	MHz	166	146
transfer resonator eff. bandwidth, $\kappa_t/2\pi$	MHz	32.0	36.2

TABLE SII. Measured device parameters for samples installed at nodes A and B.

the whole protocol of the Bell test with the exception of the readout.

As discussed in Supplementary Section VII, we flux-bias the qutrits to a frequency $\omega_q \approx 7.2$ GHz optimized for fast and high-fidelity readout. The parameters of the readout circuitry were determined at that frequency.

We also determine the thermal excitation of the qutrit at equilibrium, and conclude that the filtering of high-frequency signals on the lines may be improved in the future, e.g. by installing high-frequency infrared absorber filters [27] on the output lines, to further decrease this value.

V. EXPERIMENTAL SETUP

In this section we provide an overview of the electronic components of the experimental setup and we discuss the synchronization between the two remote nodes A and B.

A. Wiring Configuration

A scheme of the experimental setup is depicted in Fig. S5. The quantum devices (blue and red boxes at base temperature) are magnetically shielded from the environment by an aluminium shield enclosed by two cylindrical Ni-Fe-alloy shields. To further provide a low temperature radiation environment around the sample, we use a copper shield as the innermost layer. The signals enter and leave the shielded sample box through coaxial cables. A flexible coaxial microwave cable connects each sample to the WR90 superconducting aluminium waveguide used as the quantum communication channel, see Supplementary Section III.

We route the qubit charge and readout lines through a side-port, as discussed in Supplementary Section III. We use the charge line to apply microwave pulses to the qubit with a direct synthesis arbitrary waveform generator (AWG), with the signals routed through 20 dB attenuation each at the 4K, 100 mK, and base temperature stages of the dilution refrigerators. The same attenuation scheme is used for the readout probe tone input, applied through the top flange of each dilution refrigerator. The signal generated by the room temperature electronics co-propagates with excess noise and thermal radiation along the signal lines and is attenuated and re-thermalized in the attenuators installed in the experimental wiring, see Ref. 25 for details. We use flux lines to bias the qubits at the desired frequencies with DC signals attenuated by 20 dB at the 4K stage, and low-pass filtered at the base temperature stage. On all lines, we use high-frequency infrared absorber filters [27] at the base temperature stage. The readout signal is amplified using phase-preserving Josephson parametric amplifiers (JPAs) [28] with a gain of 31.7 dB at site A and 27.9 dB at B, and a 3dB-bandwidth of 9.3 MHz and 12.1 MHz, respectively. The JPAs are mounted in individual pairs of Ni-Fe-alloy shields at the base stage of the dilution refrigerator. Routing the corresponding microwave lines through the side-port, we further amplify the readout signal at the 4K stage using a high-electron mobility transistor (HEMT) amplifier. At room temperature, the signal is amplified by a set of low noise and ultra-low noise amplifiers in the warm amplifier board (WAB) and then down-converted (DCB) to an IF-frequency of 250 MHz.

B. Setup Synchronisation

In this section we discuss the synchronization between the two remote setups A and B, which is essential for the faithful reconstruction of the quantum state shared between the two qubits. The task of synchronising the room-temperature electronics consists of two parts. First, we distribute a stable reference signal between both nodes such that the devices establish stable relative phases of their internal oscillators and output signals to ascertain phase coherence of the shared Bell state. Second, we establish a common laboratory time reference frame by transmitting a square pulse from a central trigger device to the other devices, to time the control pulses and verify space-like separation between the qubits. We distribute both the frequency reference and the timing reference in a cascade starting from node C located at the center of the cryogenic link, see Fig. S5.

A Rubidium clock at node C provides a stable 10 MHz reference to which all other instruments are synchronized. The 10 MHz reference signal is connected to the reference input of a microwave generator (MWG) located at node

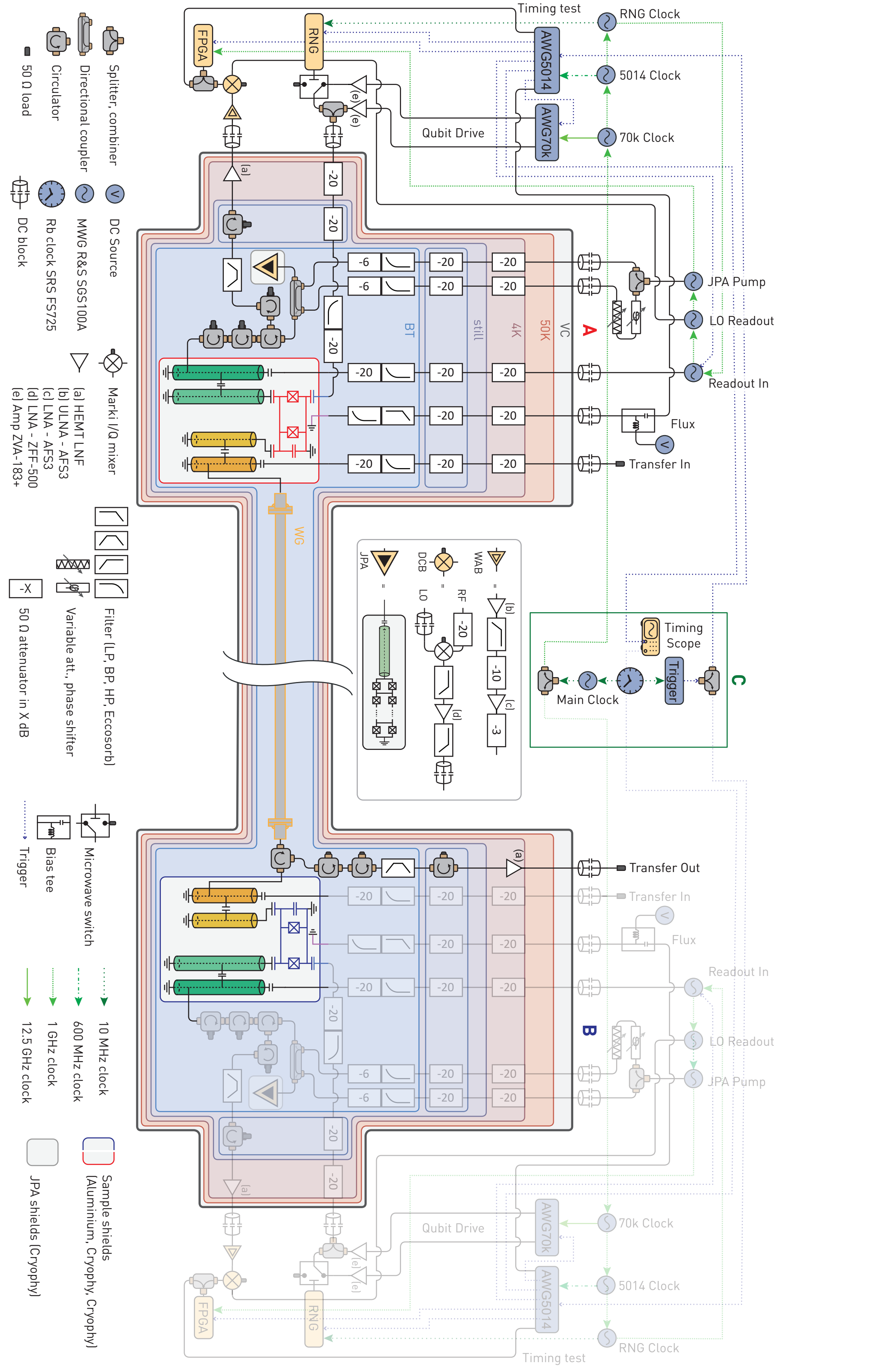

FIG. S5. Experimental setup, including control and readout of the qubits, and synchronization of the two distant setups. The elements at node B that are equivalent to the ones at node A are drawn translucent. See text for details.

C, creating an output signal at $\omega_{\rm ref}/2\pi = 1$ GHz, used as a high-frequency reference which is then distributed to nodes A and B through 20-meter-long room-temperature coaxial cables. A 1 GHz high-frequency reference signal provides better phase stability and is less affected by temperature fluctuations in the laboratory, which cause local changes of the electrical length [29], than the standard reference signals at 10 MHz. To minimize fluctuations of the electrical length due to changes of the temperature in the laboratory, we thermally isolate the coaxial cables using an elastomeric foam based on synthetic rubber. At each node, $\omega_{\rm ref}/2\pi = 1$ GHz is daisy-chained to all MWGs used to drive pulses and clock AWGs and RNGs. The local AWGs are clocked by reference signals generated by dedicated MWGs. For the Tektronix AWG5014 serving as a timing master device at each node (see below) we use a 600 MHz reference input, at the highest reference frequency supported by the device. The same AWG is used for the generation of flux pulses, triggering and gating of the other devices of the setup. For clocking the Tektronix AWG70k, which drives qubit rotations, we use a 12.5 GHz signal, provided by a dedicated MWG (see Fig. S5).

The main trigger of the setup, located at C, is provided by a Tektronix 33250A AWG. Its signal is distributed to the master devices at nodes A and B – two Tektronix AWG5014. In turn, each AWG5014 provides trigger signals to the devices used in the experiment which require triggering, including the AWG70k, qubit readout MWG, FPGA and a RNG. To provide timing information the instruments are programmed to output additional marker signals, which we use for independent timing characterization.

We synchronize the outputs of any group of signal generation devices by adjusting their output timing offsets such that their output signals arrive simultaneously through equally long cables at an oscilloscope used for verification. The absolute time t is measured relative to a master trigger. We reference the times at which the hardware outputs the generated signals to this master trigger. The FPGA detection hardware with its ADCs is referenced to the same master trigger. Applying the signal of a signal source with known timing to the input of the FPGA detection hardware, we determine the time at which the data is registered in the FPGA relative to the master trigger.

VI. CHARACTERIZATION OF STATE PREPARATION AND MASTER EQUATION SIMULATIONS

In this section, we discuss the characterization of the excitation transfer through the quantum link by sending a photon from node A and reabsorbing it at node B to calculate the transfer efficiency. In this characterization protocol, we reset the qutrits using microwave drives [30], followed by two Gaussian π pulses preparing the coupled qutrit-transfer resonator system at node A in state $|f0\rangle$, where the first state label corresponds to the qutrit state and the second to the transfer resonator Fock state. Next, we drive the $|f0\rangle \leftrightarrow |g1\rangle$ sideband transition [31, 32] to transfer the f population of the qutrit into a photon in the transfer resonator. This photon decays into the waveguide at the rate $\kappa_A/2\pi = 32.0$ MHz. We engineer the drive pulse to emit the photon with an envelope described by the mode function $f(t) \propto \mathrm{sech}(\Gamma_t/2)$ where Γ_t is the photon bandwidth. At most, the bandwidth Γ_t can be set up to the minimum of the transfer resonator coupling rates $\min[\kappa_A, \kappa_B]$; here we chose $\Gamma_t/2\pi = 20.0$ MHz because this rate led to the highest state transfer efficiency. The photon propagates in 148 ns to the opposite end of the waveguide, which is consistent with the signal delay expected from the group velocity of the waveguide. The photon then enters the transfer resonator at the other node, where we absorb it by driving B's $|f0\rangle \leftrightarrow |g1\rangle$ sideband transition, applying the time-reversed shape of B's photon emission pulse. Finally, we map the f-level of the qutrit at node B to its g-e-manifold using a π^B_{ef} pulse and we perform averaged readout of the state of the qutrit. We repeat the protocol for the truncation times τ of the $|f0\rangle \leftrightarrow |g1\rangle$ pulses indicated in Fig. S6a, and record the transfer dynamics by measuring the qutrit population in states g, e and f. We typically reach a maximum e-level population of the receiving qutrit B of $p_e \approx 67\%$, which characterizes the transfer efficiency.

To further characterize the photon emission and absorption process we measure the mean photon field in the dedicated photon detection chain, both after emitting a photon from A and B, as illustrated in the schematics of Fig. S6b and further explained in Ref. 24. We prepare the emitter qutrit in the $\frac{1}{\sqrt{2}}(|g\rangle + |f\rangle)$ state and drive the $|f0\rangle \leftrightarrow |g1\rangle$ transition to emit a photon in state $\frac{1}{\sqrt{2}}(|0\rangle + |1\rangle)$ into the waveguide. This photon has a time-symmetric envelope and a non-zero average field $\langle a_{\rm out}\rangle(t)$ proportional to its envelope.

The offset in time of the detection of the two photon envelopes (148 ns) stems from the propagation time through the 30-m-long waveguide of the photon emitted from A. We observe that the integrated photon power $\int |\langle a_{out}\rangle|^2 dt$ when emitted from A is 19.1% lower compared to the corresponding value of B's photons. This difference stems from the additional loss of A's photon in this measurement, as it passes the waveguide and circulator twice. We use this value to estimate the loss in the quantum channel for master equation simulations of the experiment. The observed value of the loss agrees with the estimate of the total insertion loss of the channel, based on the loss of its individual components, i.e. a circulator ($\approx 13\%$ loss) and two coaxial copper microwave cables with copper printed circuit boards ($\approx 3\%$ loss each).

As discussed in the main text and displayed in Figs. 2 and S6, we perform a master equation simulation of the state transfer protocol, the waveguide loss measurement,

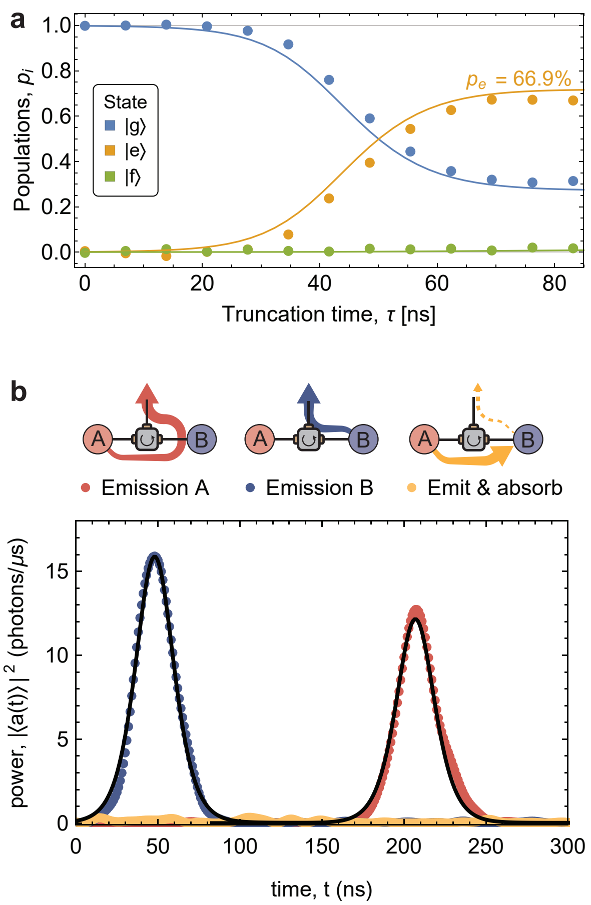

FIG. S6. a, Characterization of the transfer of a quantum state from A to B, depending on the truncation time τ of the transfer pulse. The graph shows the measured state population of the absorption qutrit at node B. The maximum transfer efficiency p_e for the excited state is 66.9%. The lines are master equation simulations. b, Measurement of the integrated power of the emitted photons (averaged over 500,000 repetitions) from A (red) and B (blue). The black lines show the results of a master equation simulation. The yellow points correspond to signatures of a photon emitted at A and absorbed at B. The y-axis is normalized to obtain a unit integrated power for photons emitted from node B.

and the entanglement generation protocol. For the master equation simulations we use the measured characteristics of the qutrits and resonators, see Table SII, as well as the measured photon loss discussed above as input values. For the state transfer protocol, see Fig. S6a, we find reasonable agreement between the measurement and the simulation, with a slightly higher transfer efficiency suggested by the simulation. We explain this difference with experimental imperfections in the emission and absorption protocol not modeled by the simulation, and synchronisation imperfections between the two setups. For the master equation simulation of the photon envelopes in the loss measurement, see Fig. S6b, we find excellent agreement between simulation and experimental data.

Finally, we simulate the entanglement generation protocol and find a simulated Bell state fidelity of 84.4%, slightly higher than the experimentally achieved value of 80.4%. Potential causes for this difference are discussed in the following. First, amplitude noise of the $|f0\rangle \leftrightarrow |g1\rangle$ sideband transition drive could be a source of dephasing. A second cause could be remaining relative phase noise stemming from synchronization imperfections between the two setups (Supplementary Section IX). Third, an unwanted, incoherent photon emission can be triggered when the $|f00\rangle \leftrightarrow |g11\rangle$ transition is activated, where the second and third state label denote the Fock state of the readout and transfer resonators coupled to the qutrit. As a consequence, the maximal S-value predicted from a master equation simulation, given the simulated Bell state fidelity, is also slightly higher ($S_{\text{sim}} \approx 2.20$) than the observed value ($S_{\text{exp}} = 2.074$), see Fig. 5 in the main text.

VII. QUBIT READOUT

In the Bell test experiment presented here, we employ the dispersive readout scheme, for which the resonator-transmon interaction causes frequency shifts of the readout probe tone depending on the state of the qubit, see Ref. [26] and references therein. We aim at a short readout duration, as it is the dominant contribution to the total protocol time budget. In addition, the experimentally achievable S-value depends quadratically on the readout fidelity, see Eq. (1) in the main text, with a Bell inequality violation requiring $\mathcal{F}_r \gtrsim 95\%$ considering the concurrence of the Bell state we achieve in the system ($\mathcal{C} = 0.765$, see Fig. 2a).

To fulfill these two stringent requirements, we tailored the parameters of qubits and readout resonators for fast, high-fidelity readout at the design stage of the quantum devices [26]. More precisely, we opted for increasing the qubit state-dependent dispersive frequency shift χ_{ge} on the readout signal by using transmon qubits with high anharmonicity ($\alpha \approx 310$ MHz) and coupling the on-chip readout resonator to a Purcell filter to suppress qubit decay [33]. At the same time, we also chose to increase the bandwidth κ of the resonator to obtain a small ratio of dispersive shift to resonator linewidth $\chi_{ge}/\kappa \approx 0.25$, which is optimal for short readout times $\tau = 50$ ns, where $\kappa\tau < 4.5$ [26].

In addition, we tune the qubit frequency from its symmetry point to an optimal readout frequency where the detuning between the qubit A (B) and its readout resonator is reduced, and therefore the dispersive shift is increased to -6.4 MHz (-8 MHz), allowing for faster and higher-fidelity readout given the effective bandwidth of the readout resonators.

More specifically, to perform readout we first apply a square pulse through a longitudinal (flux, σ_z) control line and tune transmon A (B) away from its bias point at 7.811 GHz (7.875 GHz) used during entangle-

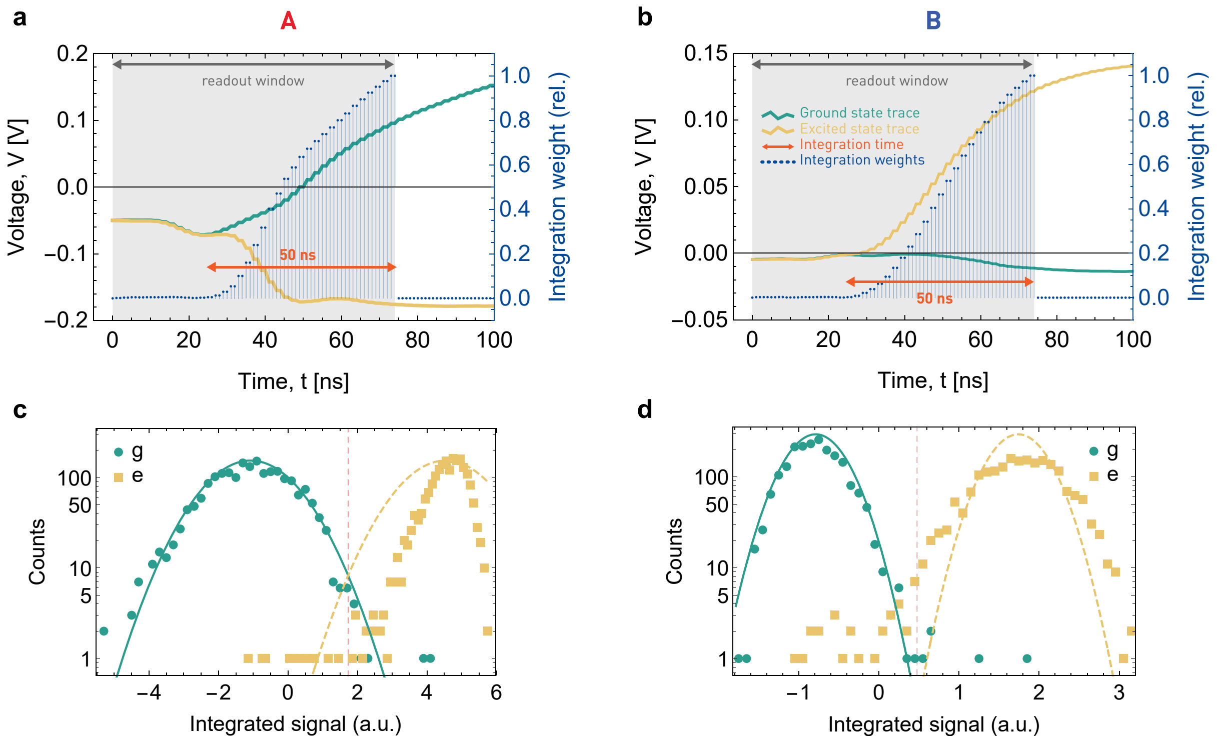

FIG. S7. Calibration and characterization of the qubit readout at sites A and B. a, b, Time traces of the Q (I) quadratures of the down-converted measurement signal of the qubits A (B) after preparation in the ground (green) or first excited state (yellow). Here we display the quadrature which contains the most information about the qubit state. The signal integration window on the FPGA is integrated in grey, beginning at the zero time in the laboratory frame (see Supplementary Sections V B and XI). The optimal integration weights are shown in dark blue. c, d, Histograms of the readout signal response of individual repetitions of the measurements in panels a and b, integrated over the indicated readout window. The solid green and dashed lines are Gaussian fits to the data. Here, the width of the excited state histogram (dashed) was fixed to the width of the ground state (solid), see main text. The vertical dashed line indicates the threshold above which the measured state is classified as excited (e), and as ground (g) below.

ment generation to the frequency optimized for the fast readout 7.200 GHz (7.550 GHz) (see Table SII). The benefit of the larger dispersive shifts outweighs the fact that both qubits have significantly reduced coherence times ($T^*_{2ge} \approx 0.3\mu$s) at these frequencies.

When the qubit is tuned to its readout frequency, we apply a gated microwave tone to the input of the readout circuitry. The readout signal is routed through two cryogenic circulators to a quantum-limited Josephson parametric amplifier (JPA) [34] located at the base stage of the respective dilution refrigerator (see Fig. S5). Biased to be used in phase-sensitive mode (on resonance with the resonator probe tone), the JPA yields about 31.7 dB (27.9 dB) amplification with a 3 dB bandwidth of 9.3 MHz (12.1 MHz). We further route the signal through a set of circulators and a band-pass filter to a high-electron-mobility transistor (HEMT) amplifier operated at the 4K stage and to the side-port of the cryostat.

At room temperature we further amplify and downconvert the signal to 250 MHz, record it with an analog to digital converter (ADC), and process it by a field-programmable gate array (FPGA) which transmits the data to a control PC [35]. For the Bell test measurements we digitally down-convert the signal to DC (which results in two signal quadratures I and Q) and perform a weighted integration of the quadrature amplitudes with calibrated weights. Using the FPGA we then threshold this integrated complex amplitude of a single time trace to determine the qubit state. The integration window on each FPGA starts at the zero time in the laboratory time frame and ends at the specified cutoff time, as discussed in Supplementary Section XI.

We calibrate the single-shot readout weights by preparing the qubit in the ground and excited state, 1000 times each, and comparing the averaged time traces as shown in Figs. S7a, b. The relative weights for each FPGA sample (sampling time of 1 ns) are set according to the complex amplitude difference of the two traces in that sample. The signal-to-noise ratio of the readout signal scales with the separation of the two readout traces under the assumption that all points are exposed to Gaussian

white noise [36]. The presented readout scheme therefore optimizes the integration in the sense that the weighting favors data points with higher signal-to-noise ratio. The weights are set to zero outside of the integration window. The calibrated state assignment thresholds and integration weights are fixed before the Bell test experiments are performed.

In a next step, we calculate the signal amplitudes of the individual single-shot traces of the measurement described above using weighted integration. We then fit this data for each quadrature using a double Gaussian model, see Fig. S7c, d for the data of the quadratures with the highest contrast between the two qubit state-dependent signal responses. Because of FPGA software limitations, we fit both Gaussian distributions with a fixed width, determined by the width of the ground state histograms. We then choose the qubit state assignment threshold as the intersection point of the Gaussian fits. In the future, one could implement a software upgrade which allows an optimized extraction of the qubit state assignment threshold by extracting the threshold solely from the measured data and not via the Gaussian fit.

From the measured data we determine a readout assignment fidelity $\mathcal{F}_r = 1 - P(e|g) - P(g|e)$ of 99.1% for node A and 97.60% for node B with a readout integration time of 50 ns at each site. This performance is similar to the one reported in Ref. 26.

We note that for all experiments discussed in this manuscript qubit reset was implemented at the beginning of each individual pulse sequence, including for characterization of the readout protocol. The reset scheme we employ is described in detail in Ref. 30, based on driving the same $|f0\rangle \leftrightarrow |g1\rangle$ transition which we also use for the generation of entanglement (Supplementary Section VI). In this continuously driven reset-scheme, population is transferred from the first two excited states of the qutrit to excitations in the transfer resonator. While we drive the reset for 1 μs, most of the population of the first two excited states of the qutrit is transferred to the resonator within the first 100 ns of the protocol. The remaining excited state population of the qubit after applying the reset scheme is below the detection threshold of our measurement scheme, which is below 1%.

We also analyzed the dependence of the readout fidelity at sites A and B on the integration time (Fig. S8) to estimate by how much the time for readout, and thus also the total duration of the Bell test protocol, could be reduced while still violating the Bell inequality. Reducing the readout integration time not only helps in reducing the total time of each Bell test trial, but it also decreases the S-value due to the lower readout fidelity achievable at a reduced integration time, also see the discussion in Supplementary Section VIII. In this analysis (Fig. S8), we use the same data as shown in Fig. S7, but including only a subset of the readout signal corresponding to reduced integration times. For each displayed integration time, we calculate the optimal integration weights and the state assignment threshold, and the corresponding readout fidelity. While the readout fidelity at A remains above 95% even when reducing the integration time to 30 ns, the corresponding fidelity at B drops much faster. We believe that the readout at B is worse due to a defect mode observed on that sample at a frequency of ∼7.4 GHz, which couples to the qubit and therefore causes additional loss of the qubit population before and during the readout.

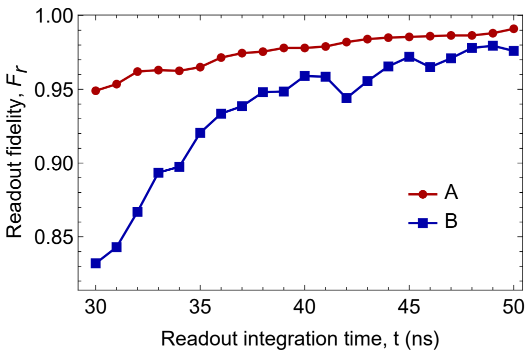

FIG. S8. Readout fidelity versus integration time for each qubit for data in Fig. S7, but only taking into account a limited set of data points, corresponding to reduced integration times.

VIII. COMPARISON TO PUBLISHED LOOPHOLE-FREE BELL TESTS

In this section we compare key aspects of our experiment to published loophole-free Bell tests. In a first part, we discuss the achieved Bell violation, repetition rate and p-value, and in a second part we discuss closing the locality loophole.

A. Bell Violation, Repetition Rate and p-value

In the following, we compare the main result and statistics of published loophole-free Bell tests and experiments on device-independent randomness generation and expansion, as well as device-independent quantum key distribution. (Some publications quoted the CH-Eberhard [45] instead of the CHSH S-value. For a more straightforward comparison we converted the respective numbers to the CHSH S-value using $S_{\mathrm{CHSH}} = 4S_{\mathrm{CH}} + 2$ both in the text and in Figure S9.) Our experiment based on superconducting circuits combines high S-values with high repetition rates. This combination allows us to reach a strong statistical significance (p-value) of $p = 10^{-108}$, and it will greatly facilitate the implementation of a variety of protocols related to device-independent quantum information processing [46–49], for which the combination of high Bell violation and high repetition rates is desirable.

We note that Bell tests which did not address all loopholes simultaneously typically reached higher S-values,

also because they were subject to weaker constraints than tests addressing all major loopholes. For instance, for experiments using single-photon detectors with limited efficiency, the conclusive events can be postselected [45, 50]. This however requires to invoke the fair sampling assumption [3], and thus opens the fair-sampling loophole. Early Bell tests using the CHSH [51] inequality, such as Ref. 52, invoked the fair sampling assumption and found an S-value of $S = 2.697 \pm 0.015$. The first experiment to close the locality loophole also relied on the fair sampling assumption and measured an S-value of $S = 2.73 \pm 0.02$ [53]. The first experiment to avoid this assumption by closing the fair-sampling loophole while not addressing other loopholes reached $S = 2.25 \pm 0.03$ [54].

B. Locality Loophole

In this section, we compare the margins with which published loophole-free Bell tests close the locality loophole. We also discuss approaches to further increase the margin of our experiment in the future.

In this context, it is important to appreciate that in the literature different criteria are used for defining the time at which the result of a quantum measurement is fixed. A variety of physical processes during the creation of a measurement result, starting from the time at which the quantum system under test interacts with the measurement apparatus to the time at which the measurement result is stored in hardware, are considered in the literature as the final event concluding a Bell test trial. An extreme view could consider the measurement process to be finished when a conscious being has appreciated the measurement outcome. Considering physical processes occurring earlier during the detection process facilitates closing the locality loophole while considering later processes makes it harder to close the locality loophole requiring larger space-like separations between the entangled particles. Generally, experiments using stop events later in the measurement chain are able to test a broader class of local realist theories than those which use earlier ones.

As discussed in the main text and in Supplementary Section VII, we consider the measurement result of a single Bell test trial to be fixed once the measurement signal corresponding to the last time bin that will be considered in the integration arrives at the input of the ADC (Fig. S11). A similar approach was used in Refs. 13, 14, and 40, where the arrival of a photon signal at a specific threshold amplitude at a digitizer or time tagger was chosen as the stop event of the Bell test trial. A more conservative approach was taken in Ref. 15, which included the digitization time of the analog signal. In our experiment, this would require waiting for additional 7 ns for the ADC to digitize the signal [57]. The Delft experiment (Ref. 12; and Ref. 37 presenting a second data set) took an even more conservative approach, including post-processing time in the digital detection

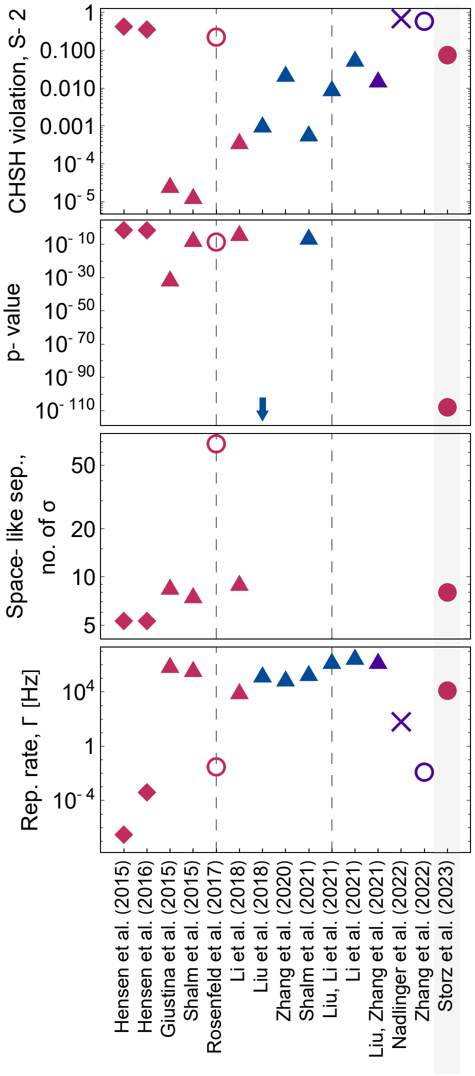

FIG. S9. Overview of key experimental metrics of Bell tests that close all major loopholes and experiments related to device-independent quantum information processing. We compare the experimentally achieved normalized CHSH inequality violation $|S|$-2 and p-value, the margin between the protocol duration and the timing budget for space-like separation expressed in numbers of standard deviations, and the data acquisition rate. The shape of the markers indicate the technological platform used for the experiment: NV-centers in diamond (rhombi), polarization-encoded photons (triangles), trapped ions (crosses), neutral atoms (open circles) and superconducting circuits (full circles). The colour of the markers indicate the type of experiment implemented: loophole-free Bell tests [12–15, 37, 40] (red), device-independent randomness generation and expansion [41–44, 55] (blue), and device-independent quantum key distribution [38, 39, 56] (violet). The dashed lines serve as a guide to the eye. We note that many of the experiments realizing device-independent quantum information processing protocols should reach a low p-value, even though it is not always explicitly quoted. In particular, the experiment presented in Ref. [41], marked with an arrow in the second plot, quotes a p-value of $p = 10^{-204792}$.

chain until the measurement was stored as a bit in a digital memory, and thereby testing an even broader set of local realist theories. With the FPGAs used in our setup, this would take about an additional 450 ns. This time consists of the parallelization of the data in the FPGA through a deserializer (~ 100 ns), the standard processing of the signal in the FPGA including filtering (~ 90 ns), the computation related to the state identification of the readout trace (~ 160 ns), and the process of writing the data to the DDR3 memory (~ 100 ns).

On the other hand, one could consider the measurement signal to be fixed already earlier, after it has passed the first or second amplification stages within the dilution refrigerator. The first amplification stage, a Josephson parametric amplifier (JPA) [58, 59], amplifies the qubit readout signal by $\sim$25 dB while adding mostly quantum-limited noise (see Supplementary Section V). We amplify the signal a second time using a high-electron-mobility transistor (HEMT) amplifier, located at the 4K stage of the dilution refrigerator. This is the first amplification (about ~ 40 dB) which is not quantum-limited, adding noise photons that are uncorrelated with the signal [58]. Taking either of these as the stop events has two effects: it reduces the protocol duration, because of a reduction in the signal propagation time, but it also decreases the Bell time budget, because of a reduction in the distance between start and the now adjusted stop event. Since we have optimized the propagation time of the time-critical signals between sample and room-temperature electronics (Supplementary Section III), the margin between the protocol duration and the Bell time budget only changes slightly if we consider earlier stop events compared to the approach described in the main text. If we consider the measurement result to be fixed once the signal has passed the second amplification stage (HEMT), the net timing margin increases by 0.57 ns. If we further argue that the measurement is completed already once the signal has been amplified in the JPA, our time margin increases by a total 2.37 ns, roughly doubling the current margin (see Table SIII).

We envision two main options to further increase the timing margins in the experiment for future implementations of non-local protocols. The first is to add extra cryogenic link modules between nodes A and B, thereby increasing the Bell distance while not affecting the protocol duration. Each additional link module increases the physical separation by 2.5 m, therefore adding ~ 8.3 ns of extra time budget. The ultimate length limit of the current setup is given by the length of the laboratory, which allows for the addition of 4 extra link modules. In principle, and supported by heat-flow simulations based on the measured thermal properties of the system, the presented cryogenic-link architecture could be extended to distances of several 100 m when placing a 4K cooling unit every 15 m and a dilution refrigerator every 100 m. Increasing the length of the cryogenic quantum channel to km-scale distances could be achieved with optimizations in the design of the system and the selection of materials. Operation of the waveguide at elevated temperatures, as suggested in [60], may also increase the maximum separation that can be achieved between link nodes. An alternative approach would be to shorten the total protocol duration, by further decreasing the measurement integration time (currently 50 ns), see Fig. S8. This however lowers the readout fidelity at the two nodes, and in turn the experimentally achievable S-value (Eq. (1) in the main text). At the moment, the readout at site B limits this fidelity. Given the measured Bell state concurrence and the readout fidelities shown in Fig. S8, we estimate with Eq. (1) in the main text that the minimal readout time required for violating Bell's inequality would be 40 ns in the current setup.

Table SIII lists Bell tests which address all major loopholes and their margins for verifying space-like separation, both absolute and relative to the Bell time budget. We compare the following quantities: first, the time budget t_d, given by the spatial distance between the start and stop events d divided by the speed of light c, $t_d = d/c$; and second, the overall critical duration of the Bell test ("Bell duration"), characterized by the time between the start and stop events $t_{\text{exp}} = t_\times - t_\star$, as well as the standard deviation of this value. From these numbers we then calculate the time margin, defined as the difference between the time budget and the Bell duration $t_{\text{margin}} = t_d - t_{\text{exp}}$, and the percentage of the margin in the total time budget, $t_{\text{margin}}/t_{\text{d}}$. Finally, we calculate the number of standard deviations that separates the Bell duration from the time budget $(t_{\text{margin}}/(\sigma_{t_d}^2 + \sigma_{t_{\text{exp}}}^2)^{1/2})$, given the stated measurement errors. This can be interpreted as a measure of confidence for closing the locality loophole. For the Bell duration and time margin of Ref. 14, we take the number representing the dataset with the lowest p-value (see Fig. S9). In this experiment, the authors also present different datasets with more and less timing margin. For Ref. 40, we take the minimal margin for run 3, which was presented as the main data set.

In this comparison we find that our experiment has similar relative time margins as Refs. 12 and 13 and slightly lower margins than Refs. 14 and 40. In terms of the statistical distance between the measured protocol duration and Bell time budget, our experiment performs at a similar level as the implementations presented in Refs. 12–14, 37, and 40. The only loophole-free Bell test with notably higher relative timing margin and statistical significance regarding closing the locality loophole is Ref. 15.

Experiment	Definition of end of trial at $t_\times$	Time budget t_d	Bell duration $t_{\text{exp}} = t_\times - t_\star$	Time margin $t_{\text{margin}} = t_d - t_{\text{exp}}$	Percentage of margin in total time budget $t_{\text{margin}}/t_{\text{d}}$	Space-like separation expressed in number of std. devs. (σ)
Storz 2022 Zurich (current work)	Trailing edge of measurement signal enters digitizer	109.48 ± 0.015 ns	107.4 ± 0.25 ns	2.08 ns	1.9%	$8.0\,\sigma$
Hensen 2015/2016 Delft [12, 37]	Result stored in classical memory	4270 ± 5 ns	$4180 + 16$ ns*	89.6 ns	2.1%	$5.3\,\Delta t_{\text{exp}}$*
Giustina 2015 Vienna [13]	Signal crosses threshold amplitude in digitizer	194.1 ± 0.3 ns	190.4 ± 0.3 ns	3.7 ns	1.9%	$8.7\,\sigma$
Shalm 2015 Colorado [14]	Signal crosses threshold amplitude in time tagger	616.8 ± 3.3 ns	578.5 ± 3.7 ns	38.3 ns	6.2%	$7.7\,\sigma$
Rosenfeld 2017 Munich [15]	Digitization of signal concluded	1324.2 ± 3.3 ns	1093 ± 1 ns	234.6 ns	17.7%	$68.0\,\sigma$
Li 2018 Shanghai [40]	Signal crosses threshold amplitude in digitizer	610.4 ns $\pm$ 3 ns	564.4 ± 4 ns	46.0 ns	7.5%	$9.2\,\sigma$

TABLE SIII. Comparison of space-time measures of loophole-free Bell tests, relevant for closing the locality loophole, see text for details. *The authors of Refs. 12 and 37 estimate a conservative upper bound $\Delta t_{\text{exp}} = 16\,\text{ns}$ on the error in t_{exp} rather than specifying a standard deviation σ on the Bell test duration. As a consequence, the corresponding statistical distance between the measured protocol duration and the Bell time budget (last column) is expressed in terms of Δt_{exp} for that experiment.

IX. PHASE STABILITY OF EXPERIMENTAL SETUP

With the control electronics of both setups separated by a distance of 30 meters we face the challenge of calibrating the relative phase of the instruments at the two nodes A and B, and to maintain phase coherence over the course of the experiment. In the following, we determine the effect of phase offsets on the measured CHSH [51] S-value and discuss the optimization of the electronic setup to keep the phase offset constant in time.

We consider a microwave pulse $b_{\text{in}}(t) = \beta_{in}(t)e^{-i(\omega_{ge}t+\phi)}$ with amplitude $\beta_{\text{in}}(t)$, frequency ω_{ge} and phase ϕ, applied to a qubit. The phase ϕ selects the axis of the Bloch sphere around which the pulse rotates the qubit state. By definition, $\phi = 0$ ($\phi = \pi/2$) corresponds to a rotation around the x (y) axis. As described in the main text, the preparation of the Bell state $|\psi^+\rangle = (|ge\rangle + |eg\rangle)/\sqrt{2}$ using the entanglement scheme presented in Ref. 24 consists of a combination of five microwave pulses: a π^A_{ge}, a $\frac{\pi}{2}{}^A_{ef}$ and an $f0g1^A$ pulse on the qubit at site A, followed by the $f0g1^B$ absorption pulse at qubit B, and a $\frac{\pi}{2}{}^B_{ef}$ rotation of qubit B. We ignore the phase of the first pulse in the following, as it only sets an initial phase on both qubits. Taking into account the phases of the other four pulses, we can write the resulting Bell state as

$$|\psi\rangle = \frac{1}{\sqrt{2}}(|eg\rangle + e^{-i(\phi^A_{ef} - \phi^A_{f0g1} + \phi^B_{f0g1} - \phi^B_{ef})}\,|ge\rangle). \quad (14)$$

We then include the measurement basis rotation and basis choice pulses at the two nodes applied just after the Bell state preparation. In combination, they can be considered microwave pulses with effective phases ϕ^A_{ge} and ϕ^B_{ge}, respectively. Combining these two offset phases with Eq. (14) and the definition of the CHSH [51] S-value $S = \langle x \cdot y\rangle_{(0,0)} - \langle x \cdot y\rangle_{(0,1)} + \langle x \cdot y\rangle_{(1,0)} + \langle x \cdot y\rangle_{(1,1)}$, we find

$$S = 2\sqrt{2}\cos\theta \quad (15)$$

which is 2π periodic in θ given by

$$\theta = \phi^A_{ef} + \phi^A_{ge} - \phi^A_{f0g1} - (\phi^B_{ef} + \phi^B_{ge} - \phi^B_{f0g1}). \quad (16)$$

In the main experiment (Fig. 5) we control the angle between the two measurement bases θ_{control} by changing the offset phase of the measurement basis choice pulse ϕ^B_{ge} at node B with respect to its counterpart ϕ^A_{ge} at node A. In practice however, the arbitrary waveform generators which produce the respective pulses at both nodes have an arbitrary phase offset. For this reason, we write $\theta = \theta_{\text{control}} + \theta_0$ where θ_0 denotes an additional, arbitrary experimental phase offset angle, which we do not have direct control over. When we present the measured S-values as a function of the experimental phase offset θ (Fig. 5), we calibrate out $\theta_0 = 160°$ by subtracting it from the experimental control parameter θ_{control}. As a result, S takes its maximum values at $\theta = -\pi/4$ and $3\pi/4$, as expected.

The second consequence is that the offset phase θ_0, albeit now characterized, fluctuates and drifts over time,

thereby again reducing the measured CHSH value. We therefore write $\theta_0 = \overline{\theta_0} + \Delta\theta_0$, describing fast oscillations represented by $\Delta\theta_0$ and slow drifts in $\overline{\theta_0}$. The phase difference fluctuations on short time scales $\Delta\theta_0$ affect the CHSH inequality within a single Bell test ($\lesssim$ 10 min) and they predominantly stem from internal phase noise of the involved instruments. The slow drifts $\overline{\theta_0}$ are problematic because they require recalibrating the optimal offset phase in between individual experiments on the time scales of tens of minutes to hours. Those drifts arise from synchronization imperfections between the instruments and their clocking signals, and from local temperature changes of cables through which reference signals are routed to the two nodes, resulting in drifts of the effective electrical lengths of the reference signal paths.

In our synchronization scheme presented in Supplementary Section V B, we have addressed these issues by choosing a clocking scheme that minimizes the internal phase noise of the instruments by using reference signals of as high frequencies as possible, and by thermally shielding the reference signal cables to prevent large low-frequency phase drifts due to local temperature variations in the laboratory. As a result, we choose to recalibrate the phase difference θ_0 roughly every hour.

X. DATA PROCESSING AND STATISTICAL ANALYSIS

In this section, we discuss the processing of the data and we present a thorough statistical analysis of the main experimental data presented in Table SV. In particular, we calculate the p-value of the experimental result, and we test if the data is compatible with the principle of no-signalling.

A. Data Processing

In the following, we discuss the post-processing of the individual trials of a Bell test. After data collection for a Bell test, the FPGAs are programmed to send a list of integers corresponding to the results for each individual Bell test trial to a central computer. These integers are converted from the input and output bits according to the assignment shown in Table SIV.

We process data collected at nodes A and B by counting the occurrences $N_{a,b,x,y}$ of the 16 possible input-output combinations and use these values to calculate

Input bit (a,b)	Meas. qubit state (x,y)	Assigned integer
0	$\vert g\rangle$ or +1	0
0	$\vert e\rangle$ or -1	1
1	$\vert g\rangle$ or +1	2
1	$\vert e\rangle$ or -1	3

TABLE SIV. Input-output bit assignment table.

Counts $N_{a,b,x,y}$	x = +1<br>y = +1	x = +1<br>y = -1	x = -1<br>y = +1	x = -1<br>y = -1
a = 0, b = 0	100,529	31,780	29,926	99,965
a = 0, b = 1	30,638	101,342	96,592	33,131
a = 1, b = 0	94,661	30,018	35,565	102,060
a = 1, b = 1	96,291	29,186	32,104	104,788

TABLE SV. Raw counts of the individual occurrences for the final Bell test for fixed offset angle $\theta = -\pi/4$ with the most statistics (2^{20} trials), presented in the main text.

the correlators

$$E(a,b) = \frac{N_{a,b,1,1} - N_{a,b,1,-1} - N_{a,b,-1,1} + N_{a,b,-1,-1}}{N_{a,b,1,1} + N_{a,b,1,-1} + N_{a,b,-1,1} + N_{a,b,-1,-1}}. \tag{17}$$

From here, we calculate the CHSH S-value [51] using the four correlators,

$$S = E(0,0) - E(0,1) + E(1,0) + E(1,1). \tag{18}$$

We explicitly note that no additional data processing, especially no correction for readout errors and no data post-selection, was performed.

For independent verification of our results we list the raw counts of the individual occurrences for the single Bell test with 2^{20} trials presented in the main text in Table SV. From this data we calculate the CHSH [51] S-value and the corresponding statistics, see Eq. (18) and Supplementary Section X B.

B. Calculation of p-value

The p-value of the Bell test is given by the probability with which any local hidden variable model could reproduce statistics at least as extreme as the results of our experiment. For the calculation of the p-value we use the description of the Bell test as a game [1], and we do not assume the individual trials to be independent of each other, in order to close the memory loophole. In a Bell game, Alice and Bob try to win as a team. In every repetition of the experiment, they each receive a question, corresponding to uniform and random input bits a and b. Alice and Bob then output a response corresponding to measurement outcomes x and y. We say that Alice and Bob win the current trial of the game if $a \wedge b = x \bigoplus y$ where $\wedge$ denotes the logical AND function and $\bigoplus$ the XOR operation. The game is repeated n times, and the number of successful ("winning") trials c is counted. Any deterministic classical strategy applied over the course of n trials yields an upper limit on the success probability of $p_{\mathrm{win}} \leq p_{\mathrm{win}}^{\mathrm{LHVM}} = 3/4 = 0.75$, while a strategy involving shared quantum entanglement allows for $p_{\mathrm{win}} \leq p_{\mathrm{win}}^{\mathrm{QM}} = \cos^2(\pi/8) \approx 0.854$.

The probability, or p-value, of a local hidden variable model yielding statistics at least as extreme as the

observed ones is given by

$$p = \sum_{i=c}^{n} C_n^i p_{\text{win}}^i (1 - p_{\text{win}})^{n-i}, \tag{19}$$

where C_n^i are binomial coefficients. This expression represents running n Bernoulli trials with success probability p_{win} resulting in at least c wins in the Bell game. It reaches a maximum at the upper bound to win for a local hidden variable model, $p_{\text{win}} = p_{\text{win}}^{\text{LHVM}} = 3/4$.

Assuming $c/n > p_{\text{win}}$ (corresponding to a Bell inequality violation), we can estimate the sum as a geometric progression. Indeed, if we define

$$t_i := C_n^i p_{\text{win}}^i (1 - p_{\text{win}})^{n-i}, \tag{20}$$

then

$$\begin{aligned} t_{i+1} &= \frac{p_{\text{win}}(n-i)}{(1-p_{\text{win}})(i+1)} \cdot t_i \\ &\leq \frac{p_{\text{win}}(n-c)}{(1-p_{\text{win}})(c+1)} \cdot t_i \end{aligned} \tag{21}$$

Note, as $p_{\text{win}} < c/n$, the prefactor is bounded by $\frac{c}{n}(n-c)/((1-\frac{c}{n})(c+1)) = c/(c+1) < 1$, and our upper bound leads to a convergent series. Therefore, the whole sum is bounded by

$$\begin{aligned} &\sum_{i=c}^{n} C_n^i p_{\text{win}}^i (1 - p_{\text{win}})^{n-i} \\ &\qquad < \frac{1}{1 - \frac{p_{\text{win}}(n-c)}{(1-p_{\text{win}})(c+1)}} \cdot C_n^c p_{\text{win}}^c (1 - p_{\text{win}})^{n-c}. \end{aligned} \tag{22}$$

Finally, using Stirling's formula, we find the following bound on the binomial coefficient C_n^c, valid for $0 < c < n$:

$$C_n^c \leq \sqrt{\frac{n}{2\pi c(n-c)}} \frac{n^n}{c^c (n-c)^{n-c}}. \tag{23}$$

Substituting this into the sum we get

$$\begin{aligned} p &= \sum_{i=c}^{n} C_n^i p_{\text{win}}^i (1 - p_{\text{win}})^{n-i} \\ &\leq \frac{1}{\sqrt{2\pi}} \frac{\sqrt{n}}{\sqrt{c}\sqrt{n-c}} \frac{\left(\frac{n p_{\text{win}}}{c}\right)^c \left(\frac{n(1-p_{\text{win}})}{n-c}\right)^{n-c}}{1 - \frac{p_{\text{win}}(n-c)}{(1-p_{\text{win}})(c+1)}}. \end{aligned} \tag{24}$$

Further substituting the maximal winning probability for classical strategies $p_{\text{win}} = p_{\text{win}}^{\text{LHVM}} = \frac{3}{4}$, the expression simplifies to

$$p \leq \frac{1}{\sqrt{2\pi}} \frac{\sqrt{n}}{\sqrt{c}\sqrt{n-c}} \frac{1}{1 - \frac{3(n-c)}{c+1}} \cdot \left(\frac{3n}{4c}\right)^c \left(\frac{n}{4(n-c)}\right)^{n-c}. \tag{25}$$

This is a simple closed analytical expression for an upper bound on the p-value.

For the final Bell test with an optimal angle θ (see main text), we performed $n = 2^{20}$ Bell trials and obtained $c = 796228$ wins in the Bell game. With these values we find $p \leq 10^{-108}$.

Finally, one can take into account a finite excess predictability ϵ of the random number generators by considering the winning probability that a local hidden variable model could achieve by taking advantage of partial knowledge of the choice of settings. Following Ref. 12, this winning probability is bounded from above by

$$p_{\text{win}} = \frac{3}{4} + 3\left(\frac{\epsilon}{2} + \left(\frac{\epsilon}{2}\right)^2\right). \tag{26}$$

For the value $\epsilon = 5.24 \times 10^{-6}$ (see Supplementary Section II), we obtain from Eq. (24) that the p-value remains below 10^{-108}.

C. No-signalling

In the following we check whether the data of the final Bell test with fixed offset angle $\theta = -\pi/4$ and with the most statistics ($n = 2^{20}$ trials) are in agreement with the principle of no-signalling through two statistical tests. In a bipartite system, if the probability of obtaining a certain measurement result at one node depends on the basis choice at the other, the system is said to be signalling. In our case, since the two nodes are space-like separated (Supplementary Section XI), a violation of the no-signalling principle would suggest that the two parties were able to exchange information faster than the speed of light, which cannot be achieved in quantum mechanics and would violate special relativity [2].

First, we perform independent checks on the four marginal statistics of the overall data reported in Table SV. Namely, we calculate the probability $p(x = 1|a, b)$ ($p(y = 1|a, b)$) for A (B) to find measurement outcome $x = 1$ ($y = 1$), given the input bits a and b. As shown in Table SVI, the probabilities p for obtaining $x = +1$ for a fixed input a are very similar for both of B's setting choices b. A similar conclusion is reached for B's measurement results conditioned on A's input bits. To provide statistical evidence of the independence of p on the setting choice of the distant party, we perform a pooled two-proportion z-test on that data, assuming the data follows a Gaussian distribution. We find that the probabilities (p-values) that data as extreme as the observed one are obtained under the null hypothesis of no-signalling are 82.6%, 34.0%, 43.5% and 20.4%, respectively, and thus the test finds no anomalous signalling effects in these data.

Second, we attempt to detect potential traces of signalling in the raw experimental data by performing an adaptive test using prediction-based ratios (PBR) [61, 62]. This method provides an upper bound on the p-value for a given model while making no assumption on the actual distribution of events. Moreover, for i.i.d. realizations, the p-value bound obtained by this method

Conditional probabilities of events	p-value of z-test
$P(x=1\|a=0,b=0)=50.4611\%$<br>$P(x=1\|a=0,b=1)=50.4612\%$	0.826
$P(x=1\|a=1,b=0)=47.532\%$<br>$P(x=1\|a=1,b=1)=47.825\%$	0.340
$P(y=1\|a=0,b=0)=49.754\%$<br>$P(y=1\|a=1,b=0)=49.647\%$	0.435
$P(y=1\|a=0,b=1)=48.616\%$<br>$P(y=1\|a=1,b=1)=48.937\%$	0.204

TABLE SVI. Probabilities of events in the main Bell test experiment with a specific outcome conditioned on the input bits at the two nodes, and the corresponding p-values of a pooled two-proportion z-test as a verification of the no-signalling principle.

is known to be asymptotically optimal. We consider here the case where the tested model consists of all no-signalling theories. Following Ref. 63, we attribute a score $r_i = R_i(a_i, b_i, x_i, y_i)$ to each measurement trial $i \leq n$, where $a_i/x_i(b_i/y_i)$ is A (B's) measurement setting/outcome observed in trial i of the experiment and $R_i(a, b, x, y)$ is a test constructed for trial i from the data observed in all previous trials as provided by the method. Using the fact that the expectation value of the tests satisfies $\langle R_i \rangle \leq 1$ for all no-signalling theories, it can be shown that the p-value of the observed data according to no-signalling theories is then upper-bounded by

$$p \leq \min\{1/t_n, 1\}, \tag{27}$$

where $t_i = \prod_{j \leq i} r_j$ quantifies the degree of incompatibility between no-signalling models and the data observed up to round i. We apply this method on the experimental data starting from round number $i_0 = 1001$, see Fig. S10 (the first 1000 measurement rounds are used to initialize the method). The final value of t_n smaller than one yields only the trivial bound of 1 on the p-value, meaning that no evidence of signalling is detected.

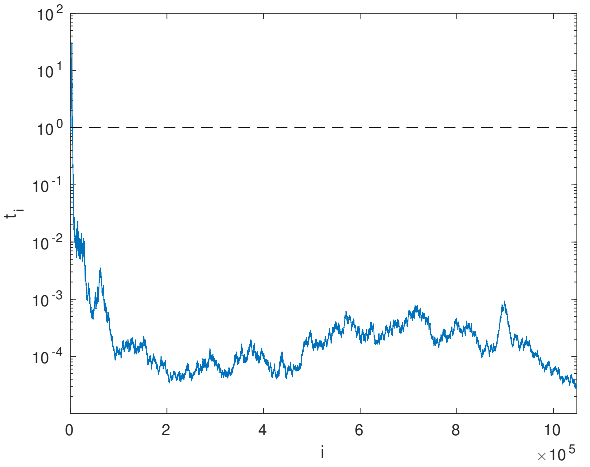

FIG. S10. Compatibility of the experimental data with the no-signalling condition along the course of the experiment. A no-signalling realization is unlikely to yield a value of t_i larger than 1. The final value of t_i allows one to compute an upper bound on the p-value of the experimental data with respect to no-signalling theories.

XI. SPACE-LIKE SEPARATION

Closing the locality loophole through space-like separation requires having precise knowledge of and control over the absolute and relative timings of the pulses and markers relevant for the time-critical part of the experiment. Here we discuss the procedure which we employed to verify space-like separation making use of the synchronization scheme presented in Supplementary Section V B.

A. Critical Path

We define the critical path as the space-time distance between the start and stop event of each trial of the Bell test. The start and stop events at the two nodes are those which must fall outside of the light cone of the corresponding events at the other party. The relevant devices defining the start and stop events of a Bell test trial in the reported experiment are the RNGs and FPGAs at nodes A and B, the spatial configuration of which in the setup is depicted in Fig. S11.

As described in the main text, the Bell test trial, and therefore the critical path, begins at nodes A and B when the local RNG starts producing a random bit. As described in Supplementary Section II, this happens when the laser diode is driven above its threshold voltage. The spatial position of this event at nodes A and B, the integrated optics card in the first RNG module, is marked with a star in Fig. S11.

We define the end event of the Bell test trial as the moment when the readout signal in the last time bin that will be considered in the integration of the signal arrives at the input of the analog to digital converter (ADC). This choice is motivated by the signal being clearly classical at this point in time after passing through multiple amplification steps. We discuss alternative approaches of choosing the event defining the end of a Bell test trial in Supplementary Section VIII.

B. Determining the Bell Time Budget

The available time budget for each trial is defined by the Bell distance, $d_{\text{Bell}} = \|\vec{r}_{\text{start}} - \vec{r}_{\text{end}}\|$, where $\vec{r}_{\text{start}}$ and $\vec{r}_{\text{end}}$ are the vectors of the spatial position of the starting and ending events of the critical path. As shown in Figs. S1 and S11, the laser diode at which the random bit first manifests is located in the transmission module of the box housing the RNG. We take the closest edge of that RNG module as the relevant starting point $\vec{r}_{\text{start}}$,

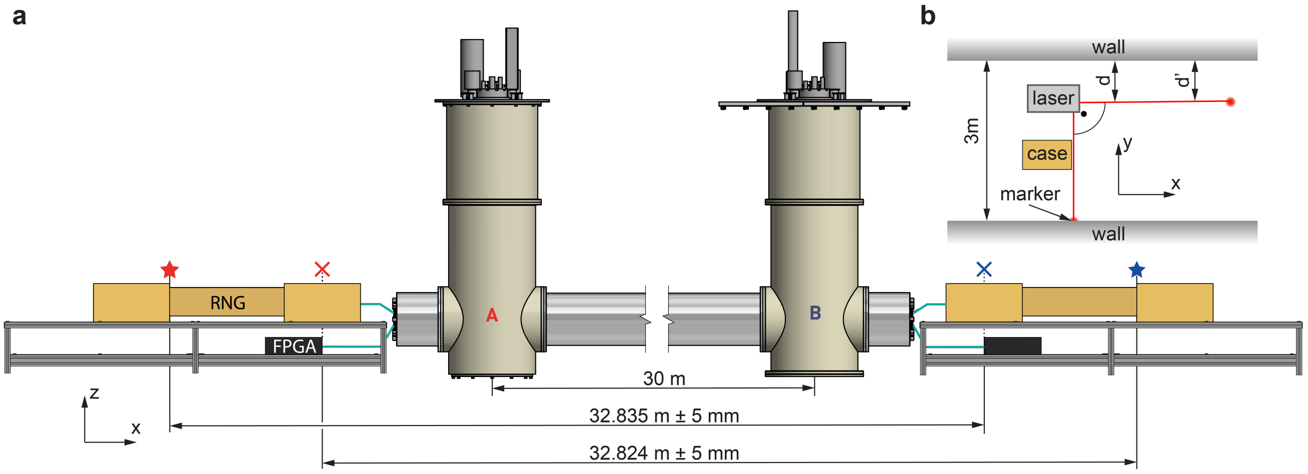

FIG. S11. Overview of the most relevant distance measures of the experimental Bell test. a, Schematic positions of the RNGs, FPGAs and dilution refrigerators at both nodes (A and B) of the setup. We indicate the measured distances between the spatial locations of the start and stop events of each Bell test trial, at the closer end of the first segment of the enclosure of the RNG, and at the input of the ADC, housed in the enclosure of the FPGA system. The star and cross markers denote the spatial position of the start and stop events of the Bell test trial. b, Top view schematic of the distance measurement procedure at one node in our laboratory using a cross line laser (red lines represent laser beams), see main text.

see star markers in Fig. S11. This is a conservative approach, as it decreases the Bell distance more than what would be needed from a purely physical point of view. Similarly, we define $\vec{r}_{\mathrm{end}}$ as the input panel of the analog to digital conversion card (ADC) on the face of the FPGA housing closest to the cryostat, see cross markers in Fig. S11.

We extract the physical distance between $\vec{r}_{\mathrm{start}}$ and $\vec{r}_{\mathrm{end}}$ from both the CAD drawing of the setup and using laser distance measurements. For this measurement, using a cross line laser (Leica Lino L2P5), we first orthogonally project $\vec{r}_{\mathrm{start}}$ and $\vec{r}_{\mathrm{end}}$ onto the notch between the floor and the wall of the laboratory (see "marker" label in Fig. S11b) as the direct line of sight between the start and stop points is blocked by the cryogenic instrumentation. We ensured that the projection is orthogonal by verifying that $|d - d'| \leq 5$mm over the distance of the whole 30-meter-long system (Fig. S11b). In a second step, we measure the distance between the orthogonally projected points $\vec{r}_{\mathrm{start}}$ and $\vec{r}_{\mathrm{end}}$ using a distance meter (Leica Disto d210) based on the multiple frequency phase-shift measurement principle. As a conservative approach, we always use the shorter of the two measured distances in the calculations related to the locality loophole, which in our setup is the distance between the start event at node B and the stop event at node A, see Fig. S11a. The shorter Bell distance measures $d_{\mathrm{Bell}} = 32,824.0 \pm 4.6$ mm. The quoted error includes the inaccuracy of the laser distance measurement and the placement of the orthogonal projection labels. As a conservative approach we neglect components of the distance in the other two spatial directions, which would only account for sub-millimeter corrections to d_{Bell}. With the speed of light of $c_0 = 299,792,458$ m/s, this Bell distance corresponds to a time budget of $t_{\mathrm{d}} = 109.489 \pm 0.015$ ns.

C. Trigger Jitter

A major constraint on the precision of the control of the timing of the various pulses is the property of digital electronics to have bi-stable input trigger regions. The arrival of a square pulse triggering the device in such a time region leads to device operation jitter in time on the order of the corresponding devices' trigger granularity. For a Tektronix AWG5014 in our setup this granularity is specified to be $6.\bar{6}$ ns, for the used FPGAs it is 8 ns and for the Tektronix AWG70000 it is 4.8 ns. Trigger jitter on this time scale adversely affects the accuracy with which we know the timing of the pulses affected by the jitter, and therefore may lead to leaving the locality loophole open. For this reason, we take measures to actively detect and subsequently avoid bistable trigger configurations.

As no method of permanently avoiding jitter caused by bistability of the trigger circuitry of the used instrumentation was known to us at the time of the execution of the experiments, we chose to carefully monitor trigger signals and make adjustments to avoid detrimental effects on the experimental data presented in the main text. For that purpose we have installed an oscilloscope at the central node C recording the arrival of auxiliary markers generated from the master devices (AWG5014s) at nodes A and B in real time (see Fig. S5). We have verified the absence of jitter from either of the AWG5014 instruments during the execution of the experiments using dedicated oscilloscopes with an effective timing reso-

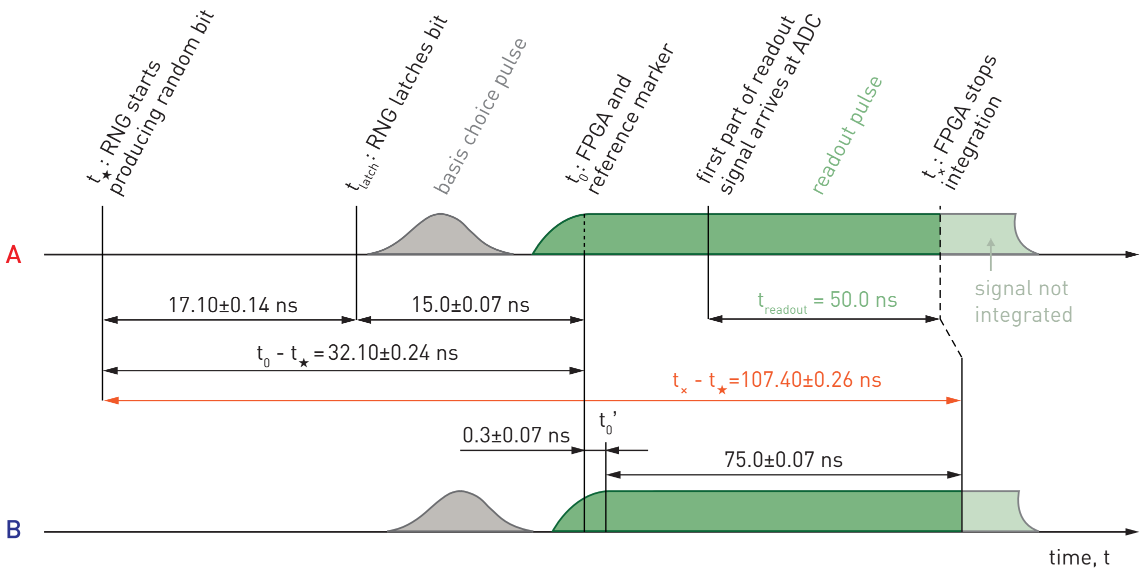

FIG. S12. Timing overview of the experiment. Measured duration and relative timings of relevant segments in the critical Bell path, as indicated by the labels. The red line corresponds to the critical protocol duration considering the start event at node A, and the stop event at node B. The graphic is not to scale.

lution of tens of picoseconds. Similarly, one oscilloscope is installed at each node to verify the absence of jitter between the devices at that node.

D. Measurement of Protocol Duration

We determine the total duration of the Bell test by summing up measurements of the duration of individual segments of the protocol. We measured the different segment durations to a precision of several hundred picoseconds. We choose the time at which the FPGA at node A starts acquiring data as our laboratory reference time t_0 for all experiments. Since we do not have experimental access to the exact time of the start event $t_\star$, we instead measure with an oscilloscope the time in the laboratory frame at which the RNG latches the random bit, i.e. when its output voltage drops to -3.5 V in case of the random bit being "1", see Fig. S1d. We perform this measurement with an oscilloscope, right before we perform the Bell test. Relative to the reference time t_0, the RNG reaches this voltage level 15±0.07 ns earlier, at t_{latch}, see Fig. S12. We choose this particular voltage reference level, since it is also used as a reference in an individual measurement to infer the random number generation time (17.10±0.14 ns, see Supplementary Section II). In combination, the duration between the start event $t_\star$ and the beginning of the FPGA acquisition window, which is our reference time t_0, is determined to be $t_0 - t_\star$ = 32.10±0.24 ns, as shown in Fig. S12. While the two setups (A,B) are synchronized in time, there is a small absolute time offset between the times the two setups consider as the reference time t_0, t_0'. This difference is caused by a small phase offset between the reference clock signals arriving at the local MWGs responsible for clocking the local AWG5014s, and it is relevant for determining the FPGA integration start time. We measure this offset with an oscilloscope to be $t_0' - t_0 = 0.30 \pm 0.07$ ns, also indicated in Fig. S12.

In order to stay well within the given time budget, we stop the FPGA integration at node B after an integration time of $t_\times - t_0' = 75$ ns. We note that the first part of the readout signal only reaches the FPGA after a propagation delay of 25 ns, and the effective readout time is therefore 50 ns (see Supplementary Section VII and Figs. S7 and S12). Outside of this integration window (i.e. for signals arriving at the input of the ADC after the chosen stop time of 75 ns) we set the FPGA integration weights to zero, such that no signal arriving outside of this window is processed on the FPGA for determining the measurement outcome. In total, we find a critical protocol duration, considering the start event at node A and the stop event at node B, of $t_{\text{exp}} = t_\times - t_\star = 107.40$ ns. We use this value for the calculation of the timing statistics (see below). For completeness we note that the protocol duration in the other direction from B to A is 0.6 ns shorter, as the synchronization offset $t_0' - t_0 = 0.3$ ns is to be subtracted for this direction, instead of added.

In addition to the measurement uncertainties of the individual segment durations, there is uncertainties in the synchronization of the two setups ($\sigma_{t_0'-t_0} = 0.07$ ns) and the synchronization between the laboratory reference frame and the local FPGA ($\sigma_{t_\times} = 0.07$ ns). In combination, they yield a measurement uncertainty of

$\sigma_{t_{exp}} = \sqrt{\sigma^2_{t_\star} + \sigma^2_{t_{\rm latch}} + \sigma^2_{t'_0 - t_0} + \sigma^2_{t_\times}} = 0.26$ ns on the total protocol duration.

From the arguments presented in this section we conclude that each trial of the Bell test has space-like separation verified, and that, therefore, the locality loophole closed, with a statistical margin of $t_{\rm margin}/\sqrt{\sigma^2_{t_{\rm d}} + \sigma^2_{t_{\rm exp}}} = 8.0$ standard deviations, where we have defined the time margin as the difference between the protocol duration and the Bell time budget, $t_{\rm margin} = t_{\rm d} - t_{\rm exp}$. This value is similar to what previously published Bell tests which closed all major loopholes reported, see Supplementary Section VIII for more details.

We verify that the locality loophole is closed before, during and after each Bell test experiment using three steps. Before starting the data acquisition for a Bell test, we perform a full timing verification procedure using oscilloscopes, auxiliary markers and test pulses sent to FPGAs at each node to ensure all devices are synchronized to the laboratory frame, and therefore the start and stop events are well known in time. We then proceed to perform the pre-defined protocol for the Bell test, during which we acquire the data presented in Fig. 5 and a single high-statistics point at an optimum measurement basis offset angle θ. While acquiring the data we monitor in real time the consistency of synchronisation and the absence of trigger jitter with three oscilloscopes as described above. Finally, we repeat a full timing verification procedure after the set of Bell test experiments. If both procedures yield the expected synchronization status and no signs of jitter are observed, we conclude the experiments to be valid and proceed to analysing their data.

[1] P. Bierhorst, A rigorous analysis of the clauser–horne–shimony–holt inequality experiment when trials need not be independent, Foundations of Physics **44**, 736 (2014).

[2] N. Brunner, D. Cavalcanti, S. Pironio, V. Scarani, and S. Wehner, Bell nonlocality, Rev. Mod. Phys. **86**, 419 (2014).

[3] J.-A. Larsson, Loopholes in Bell inequality tests of local realism, J. Phys. A: Math. Theor. **47**, 424003 (2014).

[4] J. S. Bell *Speakable and Unspeakable in Quantum Mechanics: Collected Papers on Quantum Philosophy, Chapter 24: La nouvelle cuisine*, 232-248 2nd ed. (Cambridge University Press, 2004).

[5] G. Pütz, D. Rosset, T. J. Barnea, Y.-C. Liang, and N. Gisin, Arbitrarily small amount of measurement independence is sufficient to manifest quantum nonlocality, Phys. Rev. Lett. **113**, 190402 (2014).

[6] S. Pironio, Random 'choices' and the locality loophole, (2015), arXiv:1510.00248.

[7] T. Norsen, Against 'realism', Foundations of Physics **37**, 311 (2007).

[8] N. Gisin, Non-realism: Deep thought or a soft option?, Foundations of Physics **42**, 80 (2012).

[9] T. Maudlin, What Bell did, Journal of Physics A: Mathematical and Theoretical **47**, 424010 (2014).

[10] F. Laudisa, How and when did locality become 'local realism'? a historical and critical analysis (1963-1978), arXiv:2205.05452 (2022).

[11] H. M. Wiseman, The two Bell's theorems of John Bell, Journal of Physics A: Mathematical and Theoretical **47**, 424001 (2014).

[12] B. Hensen, H. Bernien, A. E. Dreau, A. Reiserer, N. Kalb, M. S. Blok, J. Ruitenberg, R. F. L. Vermeulen, R. N. Schouten, C. Abellan, W. Amaya, V. Pruneri, M. W. Mitchell, M. Markham, D. J. Twitchen, D. Elkouss, S. Wehner, T. H. Taminiau, and R. Hanson, Loophole-free Bell inequality violation using electron spins separated by 1.3 kilometres, Nature **526**, 682 (2015).

[13] M. Giustina, M. A. M. Versteegh, S. Wengerowsky, J. Handsteiner, A. Hochrainer, K. Phelan, F. Steinlechner, J. Kofler, J.-A. Larsson, C. Abellán, W. Amaya, V. Pruneri, M. W. Mitchell, J. Beyer, T. Gerrits, A. E. Lita, L. K. Shalm, S. W. Nam, T. Scheidl, R. Ursin, B. Wittmann, and A. Zeilinger, Significant-loophole-free test of Bell's theorem with entangled photons, Phys. Rev. Lett. **115**, 250401 (2015).

[14] L. K. Shalm, E. Meyer-Scott, B. G. Christensen, P. Bierhorst, M. A. Wayne, M. J. Stevens, T. Gerrits, S. Glancy, D. R. Hamel, M. S. Allman, K. J. Coakley, S. D. Dyer, C. Hodge, A. E. Lita, V. B. Verma, C. Lambrocco, E. Tortorici, A. L. Migdall, Y. Zhang, D. R. Kumor, W. H. Farr, F. Marsili, M. D. Shaw, J. A. Stern, C. Abellán, W. Amaya, V. Pruneri, T. Jennewein, M. W. Mitchell, P. G. Kwiat, J. C. Bienfang, R. P. Mirin, E. Knill, and S. W. Nam, Strong loophole-free test of local realism, Phys. Rev. Lett. **115**, 250402 (2015).

[15] W. Rosenfeld, D. Burchardt, R. Garthoff, K. Redeker, N. Ortegel, M. Rau, and H. Weinfurter, Event-ready Bell test using entangled atoms simultaneously closing detection and locality loopholes, Phys. Rev. Lett. **119**, 010402 (2017).

[16] C. Abellán, W. Amaya, D. Mitrani, V. Pruneri, and M. W. Mitchell, Generation of fresh and pure random numbers for loophole-free Bell tests, Phys. Rev. Lett. **115**, 250403 (2015).

[17] A. Rukhin, J. Soto, J. Nechvatal, M. Smid, E. Barker, S. Leigh, M. Levenson, M. Vangel, D. Banks, N. Heckert, J. Dray, and S. Vo, A statistical test suite for random and pseudorandom number generators for cryptographic applications, NIST Special Publication, 800 (2001).

[18] M. Santha and U. V. Vazirani, Generating quasi-random sequences from semi-random sources, Journal of Computer and System Sciences **33**, 75 (1986).

[19] D.E. Koh, M.J.W. Hall, Setiawan, J.E. Pope, C. Marletto, A. Kay, V. Scarani and A. Ekert, Effects of Reduced Measurement Independence on Bell-Based Randomness Expansion, Phys. Rev. Lett. **109**, 160404 (2012).

[20] J.-D. Bancal, Analysis of a Bell test when the setting choices can be correlated to the hidden variable, Publicly available Python script: https://github.com/jdbancal/BellAtHome (2022).

[21] C. Abellán, W. Amaya, M. Jofre, M. Curty, A. Acín,

J. Capmany, V. Pruneri, and M. W. Mitchell, Ultra-fast quantum randomness generation by accelerated phase diffusion in a pulsed laser diode, Opt. Express **22**, 1645 (2014).
[22] F. Pobell, *Matter and Methods at Low Temperatures* (Springer, 3rd edition, 2006).
[23] P. Magnard, S. Storz, P. Kurpiers, J. Schär, F. Marxer, J. Lütolf, T. Walter, J.-C. Besse, M. Gabureac, K. Reuer, A. Akin, B. Royer, A. Blais, and A. Wallraff, Microwave quantum link between superconducting circuits housed in spatially separated cryogenic systems, Phys. Rev. Lett. **125**, 260502 (2020).
[24] P. Kurpiers, P. Magnard, T. Walter, B. Royer, M. Pechal, J. Heinsoo, Y. Salathé, A. Akin, S. Storz, J.-C. Besse, S. Gasparinetti, A. Blais, and A. Wallraff, Deterministic quantum state transfer and remote entanglement using microwave photons, Nature **558**, 264 (2018).
[25] S. Krinner, S. Storz, P. Kurpiers, P. Magnard, J. Heinsoo, R. Keller, J. Lütolf, C. Eichler, and A. Wallraff, Engineering cryogenic setups for 100-qubit scale superconducting circuit systems, EPJ Quantum Technology **6**, 2 (2019).
[26] T. Walter, P. Kurpiers, S. Gasparinetti, P. Magnard, A. Potočnik, Y. Salathé, M. Pechal, M. Mondal, M. Oppliger, C. Eichler, and A. Wallraff, Rapid, high-fidelity, single-shot dispersive readout of superconducting qubits, Phys. Rev. Appl. **7**, 054020 (2017).
[27] D. F. Santavicca and D. E. Prober, Impedance-matched low-pass stripline filters, Meas. Sci. Technol. **19**, 087001 (2008).
[28] C. Eichler, Y. Salathe, J. Mlynek, S. Schmidt, and A. Wallraff, Quantum-limited amplification and entanglement in coupled nonlinear resonators, Phys. Rev. Lett. **113**, 110502 (2014).
[29] R. K. Eby and K. M. Sinnott, Transitions and relaxations in polytetrafluoroethylene, Journal of Applied Physics **32**, 1765 (1961).
[30] P. Magnard, P. Kurpiers, B. Royer, T. Walter, J.-C. Besse, S. Gasparinetti, M. Pechal, J. Heinsoo, S. Storz, A. Blais, and A. Wallraff, Fast and unconditional all-microwave reset of a superconducting qubit, Phys. Rev. Lett. **121**, 060502 (2018).
[31] S. Zeytinoğlu, M. Pechal, S. Berger, A. A. Abdumalikov Jr., A. Wallraff, and S. Filipp, Microwave-induced amplitude- and phase-tunable qubit-resonator coupling in circuit quantum electrodynamics, Phys. Rev. A **91**, 043846 (2015).
[32] M. Pechal, L. Huthmacher, C. Eichler, S. Zeytinoğlu, A. A. Abdumalikov Jr., S. Berger, A. Wallraff, and S. Filipp, Microwave-controlled generation of shaped single photons in circuit quantum electrodynamics, Phys. Rev. X **4**, 041010 (2014).
[33] E. A. Sete, J. M. Martinis, and A. N. Korotkov, Quantum theory of a bandpass Purcell filter for qubit readout, Phys. Rev. A **92**, 012325 (2015).
[34] C. Eichler and A. Wallraff, Controlling the dynamic range of a Josephson parametric amplifier, EPJ Quantum Technology **1**, 2 (2014).
[35] Y. Salathé, P. Kurpiers, T. Karg, C. Lang, C. K. Andersen, A. Akin, S. Krinner, C. Eichler, and A. Wallraff, Low-latency digital signal processing for feedback and feedforward in quantum computing and communication, Phys. Rev. Appl. **9**, 034011 (2018).
[36] J. Gambetta, W. A. Braff, A. Wallraff, S. M. Girvin, and R. J. Schoelkopf, Protocols for optimal readout of qubits using a continuous quantum nondemolition measurement, Phys. Rev. A **76**, 012325 (2007).
[37] B. Hensen, N. Kalb, M. S. Blok, A. E. Dréau, A. Reiserer, R. F. L. Vermeulen, R. N. Schouten, M. Markham, D. J. Twitchen, K. Goodenough, D. Elkouss, S. Wehner, T. H. Taminiau, and R. Hanson, Loophole-free bell test using electron spins in diamond: second experiment and additional analysis, Scientific Reports **6**, 30289 (2016).
[38] W. Zhang, T. van Leent, K. Redeker, R. Garthoff, R. Schwonnek, F. Fertig, S. Eppelt, W. Rosenfeld, V. Scarani, C. C.-W. Lim, and H. Weinfurter, A device-independent quantum key distribution system for distant users, Nature **607**, 687 (2022).
[39] D. P. Nadlinger, P. Drmota, B. C. Nichol, G. Araneda, D. Main, R. Srinivas, D. M. Lucas, C. J. Ballance, K. Ivanov, E. Y.-Z. Tan, P. Sekatski, R. L. Urbanke, R. Renner, N. Sangouard, and J.-D. Bancal, Experimental quantum key distribution certified by bell's theorem, Nature **607**, 682 (2022).
[40] M.-H. Li, C. Wu, Y. Zhang, W.-Z. Liu, B. Bai, Y. Liu, W. Zhang, Q. Zhao, H. Li, Z. Wang, L. You, W. J. Munro, J. Yin, J. Zhang, C.-Z. Peng, X. Ma, Q. Zhang, J. Fan, and J.-W. Pan, Test of local realism into the past without detection and locality loopholes, Phys. Rev. Lett. **121**, 080404 (2018).
[41] Y. Liu, Q. Zhao, M.-H. Li, J.-Y. Guan, Y. Zhang, B. Bai, W. Zhang, W.-Z. Liu, C. Wu, X. Yuan, H. Li, W. J. Munro, Z. Wang, L. You, J. Zhang, X. Ma, J. Fan, Q. Zhang, and J.-W. Pan, Device-independent quantum random-number generation, Nature **562**, 548 (2018).
[42] W.-Z. Liu, M.-H. Li, S. Ragy, S.-R. Zhao, B. Bai, Y. Liu, P. J. Brown, J. Zhang, R. Colbeck, J. Fan, Q. Zhang, and J.-W. Pan, Device-independent randomness expansion against quantum side information, Nature Physics **17**, 448 (2021).
[43] L. K. Shalm, Y. Zhang, J. C. Bienfang, C. Schlager, M. J. Stevens, M. D. Mazurek, C. Abellán, W. Amaya, M. W. Mitchell, M. A. Alhejji, H. Fu, J. Ornstein, R. P. Mirin, S. W. Nam, and E. Knill, Device-independent randomness expansion with entangled photons, Nature Physics **17**, 452 (2021).
[44] M.-H. Li, X. Zhang, W.-Z. Liu, S.-R. Zhao, B. Bai, Y. Liu, Q. Zhao, Y. Peng, J. Zhang, Y. Zhang, W. J. Munro, X. Ma, Q. Zhang, J. Fan, and J.-W. Pan, Experimental realization of device-independent quantum randomness expansion, Phys. Rev. Lett. **126**, 050503 (2021).
[45] P. H. Eberhard, Background level and counter efficiencies required for a loophole-free Einstein-Podolsky-Rosen experiment, Phys. Rev. A **47**, R747 (1993).
[46] A. Acín, N. Brunner, N. Gisin, S. Massar, S. Pironio, and V. Scarani, Device-independent security of quantum cryptography against collective attacks, Phys. Rev. Lett. **98**, 230501 (2007).
[47] R. Colbeck and R. Renner, Free randomness can be amplified, Nat. Phys. **8**, 450 (2012).
[48] M. Kessler and R. Arnon-Friedman, Device-independent randomness amplification and privatization, IEEE Journal on Selected Areas in Information Theory **1**, 568-584 (2020).
[49] J. Barrett, L. Hardy, and A. Kent, No signalling and Quantum Key Distribution, Phys. Rev. Lett. **95**, 010503 (2005).
[50] A. Garg and N. D. Mermin, Detector inefficiencies in the Einstein-Podolsky-Rosen experiment, Phys. Rev. D **35**,

3831 (1987).

[51] J. F. Clauser, M. A. Horne, A. Shimony, and R. A. Holt, Proposed experiment to test local hidden-variable theories, Phys. Rev. Lett. **23**, 880 (1969).

[52] A. Aspect, J. Dalibard, and G. Roger, Experimental test of Bell's inequalities using time- varying analyzers, Phys. Rev. Lett. **49**, 1804 (1982).

[53] G. Weihs, T. Jennewein, C. Simon, H. Weinfurter, and A. Zeilinger, Violation of Bell's inequality under strict Einstein locality conditions, Phys. Rev. Lett. **81**, 5039 (1998).

[54] Rowe, Kielpinski, Meyer, Sackett, Itano, Monroe, and Wineland, Experimental violation of a Bell's inequality with efficient detection, Nature **409**, 791 (2001).

[55] Y. Zhang, L. K. Shalm, J. C. Bienfang, M. J. Stevens, M. D. Mazurek, S. W. Nam, C. Abellán, W. Amaya, M. W. Mitchell, H. Fu, C. A. Miller, A. Mink, and E. Knill, Experimental low-latency device-independent quantum randomness, Phys. Rev. Lett. **124**, 010505 (2020).

[56] W. Liu, Y. Zhang, Y. Zhen, M. Li, Y. Liu, J. Fan, F. Xu, Q. Zhang, and J. Pan, Toward a Photonic Demonstration of Device-Independent Quantum Key Distribution, Phys. Rev. Lett. **129**, 050502 (2022).

[57] T. Instruments, Technical datasheet 12-bit, 1Gsps analog-to-digital converter ads5400-sp https://www.ti.com/lit/ds/symlink/ads5400-sp.pdf (2010).

[58] C. Eichler, *Experimental Characterization of Quantum Microwave Radiation and its Entanglement with a Superconducting Qubit*, Ph.D. thesis, ETH Zurich (2013).

[59] M. Hatridge, S. Shankar, M. Mirrahimi, F. Schackert, K. Geerlings, T. Brecht, K. M. Sliwa, B. Abdo, L. Frunzio, S. M. Girvin, R. J. Schoelkopf, and M. H. Devoret, Quantum back-action of an individual variable-strength measurement, Science **339**, 178 (2013).

[60] Z.-L. Xiang, M. Zhang, L. Jiang, and P. Rabl, Intracity quantum communication via thermal microwave networks, Physical Review X **7**, 011035 (2017).

[61] Y. Zhang, S. Glancy, and E. Knill, Asymptotically optimal data analysis for rejecting local realism, Phys. Rev. A **84**, 062118 (2011).

[62] Y. Zhang, S. Glancy, and E. Knill, Efficient quantification of experimental evidence against local realism, Phys. Rev. A **88**, 052119 (2013).

[63] Y.-C. Liang and Y. Zhang, Bounding the plausibility of physical theories in a device-independent setting via hypothesis testing, Entropy **21**, 185 (2019).
